# Preoperative CT-Derived Stone Burden Metrics for Predicting Stone-Free Status and Operative Time After Retrograde Intrarenal Surgery

**DOI:** 10.3390/diagnostics16182994

**Published:** 2026-09-16

**Authors:** Shiwei Sun, Jiang Liu, Yi Qiao, Dong Wang, Hongjun Li, He Xiao

**Affiliations:** Department of Urology, Peking Union Medical College Hospital, Chinese Academy of Medical Sciences & Peking Union Medical College, Beijing 100730, China; sunshiwei71@163.com (S.S.); 2021283030155@whu.edu.cn (J.L.); fozu067504@126.com (Y.Q.); wangdong53@pumch.cn (D.W.)

**Keywords:** retrograde intrarenal surgery, urolithiasis, non-contrast computed tomography, stone burden, stone-free rate, cumulative stone diameter, risk stratification, operative time

## Abstract

**Objectives**: Non-contrast computed tomography (NCCT) is used before retrograde intrarenal surgery (RIRS) to describe stone size, location, and complexity, but the best way to summarize stone burden remains unsettled. Therefore, we compared multidimensional NCCT-derived burden metrics with cumulative stone diameter (CSD) with respect to their ability to predict stone-free rate (SFR) and operative time after RIRS. **Methods**: We reviewed 352 RIRSs performed by one surgical team from April 2024 to May 2026. The primary SFR endpoint was defined as no residual fragments or a maximum residual fragment size of ≤4 mm, with 1-month computed tomography (CT) used as the reference imaging modality. Strict 0 mm CT stone-free status was analyzed as a sensitivity endpoint. The candidate predictors included one-dimensional, two-dimensional, estimated three-dimensional, spatial-distribution, attenuation, and distribution-complexity metrics extracted from preoperative NCCT. The cohort was split into training (*n* = 247) and validation (*n* = 105) sets. **Results**: The main model retained area-equivalent diameter, staghorn stone, and stone volume distribution entropy, with a validation area under the receiver operating characteristic curve (AUC) of 0.779. A simpler CSD model combining CSD and staghorn stone achieved a validation AUC of 0.761, without a significant AUC difference (*p* = 0.201). In repeated patient-level full-pipeline validation, the median validation AUC was 0.761. Under the strict 0 mm CT endpoint, the validation AUC was 0.670. Entropy showed lower selection stability in sensitivity analyses. Reclassification favored the main model (validation continuous net reclassification improvement [NRI] 0.460, *p* = 0.007; integrated discrimination improvement [IDI] 0.038, *p* = 0.003). Regarding operative time, area-, volume-, and surface-area-based metrics performed better than CSD in regard to both the whole cohort and stone-free episodes. **Conclusions**: NCCT-derived burden metrics can refine preoperative assessment before RIRS, but their incremental value over CSD for binary SFR prediction was modest. CSD remains a practical approximation for SFR prediction, whereas multidimensional metrics better reflect operative workload.

## 1. Introduction

For a patient with urinary stones, non-contrast computed tomography (NCCT) is the map on which the operation is planned. It shows where the stones are, how many there are, how large they appear to be, whether the anatomy is favorable, and how dense the stones may be. These features influence whether retrograde intrarenal surgery (RIRS) will be chosen and how confidently stone clearance can be expected [1].

The common language of stone burden is still linear. Maximum stone diameter and cumulative stone diameter (CSD) can be quickly measured and easily communicated and are consistent with guideline-based size stratification [1,2]. Yet, the simplification is obvious in the edge cases: several small stones scattered across calyces, a thin elongated stone, a broad flattened stone, or a branching staghorn calculus may have similar summed diameters but very different endoscopic requirements. Measurement itself is not immune to variation, because CT stone size changes with plane and method [3].

Stone-level CT measurements facilitate more detailed description. Long and short axes can be translated into estimated area, volume, surface area, attenuation-weighted measures, and descriptors of how stone volume is distributed. Recent work from the European Association of Urology (EAU) Young Academic Urologists Urolithiasis Working Group and the EAU Urolithiasis Guidelines Panel supports using the added information carried by area and volume measures as opposed to a single linear diameter [4]. The unresolved clinical issue is when this added information matters enough to change predictions.

RIRS provides a useful test case. Existing scores and nomograms already show that SFRs after RIRS depend on the number and sizes of stones, lower-pole location, calyceal anatomy, density, and anatomical complexity [5,6]. What is less clear is whether stone-level distribution adds information beyond these familiar descriptors. Operative time is informative in this regard because it captures work that SFRs compress into a yes-or-no endpoint: fragmentation, inspection, relocation, basketing, and final clearance. Previous RIRS data have closely linked stone volume to operative time [7].

We therefore examined a broad set of preoperative CT-derived stone burden metrics in a single-team RIRS cohort. We hypothesized that multidimensional CT-derived metrics would refine prediction of both SFR and operative time compared with CSD, while CSD would remain a practical simplified comparator. The primary objective was to build and validate a model for the SFR and compare it with a simpler CSD-based model. The secondary objective was to identify which burden metrics best explained operative time in the whole cohort and in episodes that ended in a stone-free manner.

## 2. Materials and Methods

### 2.1. Study Design and Population

**Figure 1:**
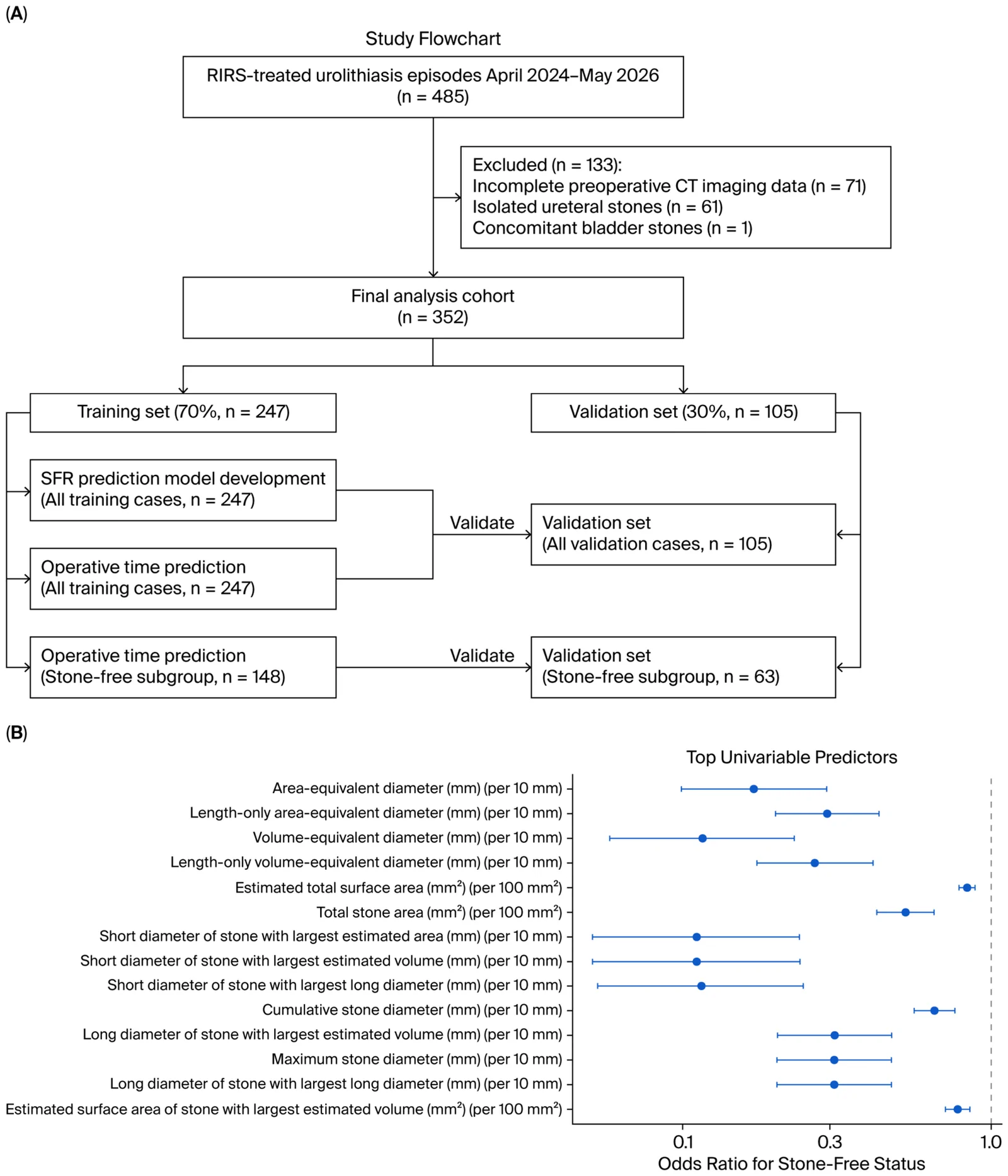

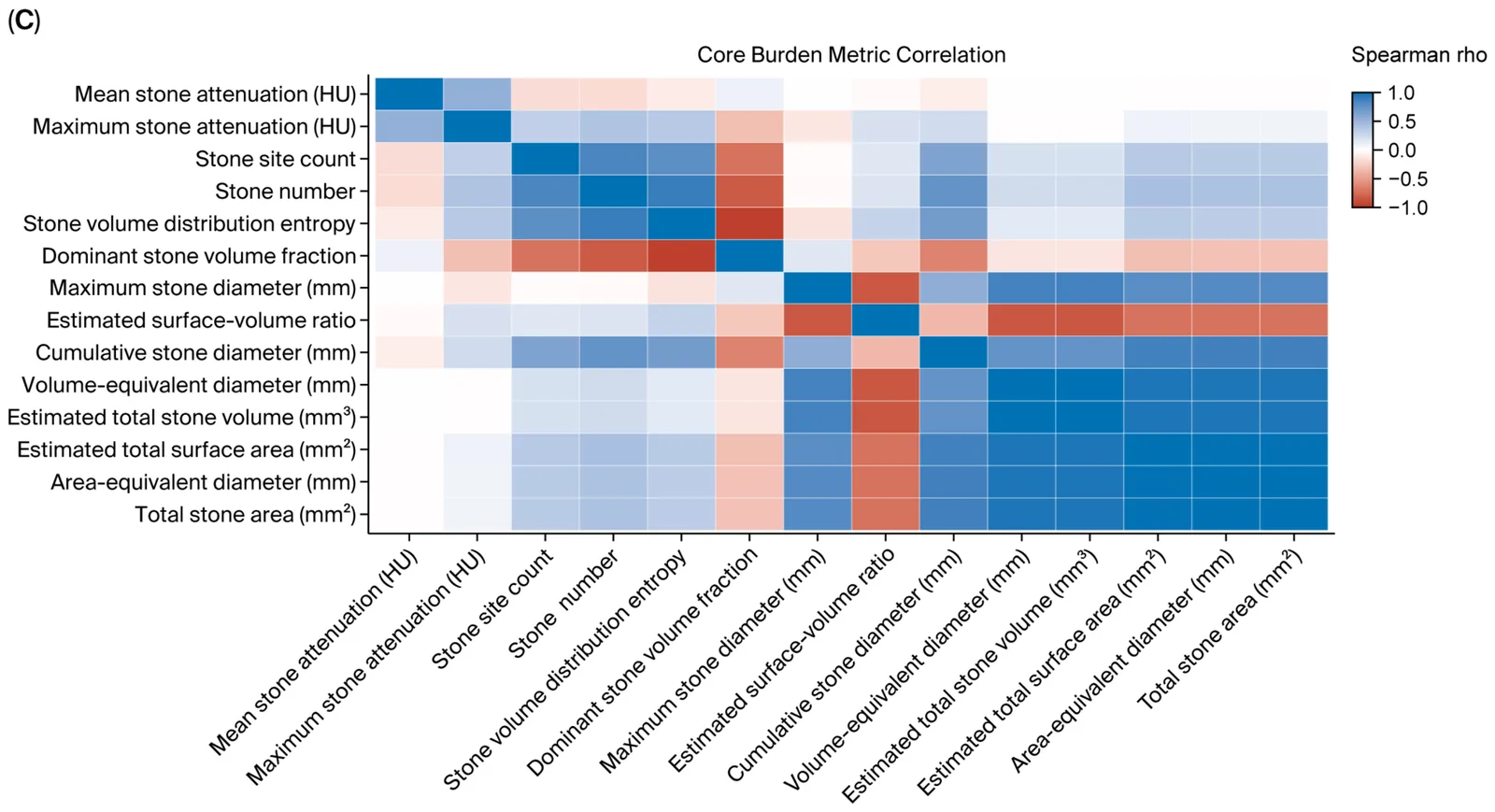
Study flow and candidate predictor screening: (**A**) flowchart of this study; (**B**) univariable associations between selected stone burden metrics and stone-free status; and (**C**) Spearman’s correlation heatmap of core CT-derived stone burden metrics.

This was a single-center retrospective cohort study. We screened 485 stone surgery episodes treated with retrograde intrarenal surgery (RIRS) by the same surgical team and the same lead surgeon between April 2024 and May 2026. After excluding 71 episodes with incomplete preoperative CT imaging data, 61 with isolated ureteral stones, and 1 with concomitant bladder stones, we included 352 episodes in the final analysis. A flowchart of this study is shown in Figure 1A. Because we conducted a retrospective cohort study, the sample size was determined by the number of consecutive eligible RIRS episodes during the predefined study period.

The unit of analysis was the surgery episode. Thirty patients contributed two RIRS episodes during the study period. Each procedure was treated as a separate surgical episode, with its own preoperative imaging, operative record, and postoperative imaging-based endpoint assessment. Eligible episodes involved RIRS for renal or upper-tract stone disease and available preoperative NCCT suitable for stone-level measurements. The exclusion criteria were as follows: having incomplete preoperative CT imaging data, isolated ureteral stones, concomitant bladder stones, or stone-level records insufficient for calculating the burden metrics defined in this study.

### 2.2. Outcomes

For the primary outcome and reference endpoint for prediction, we used postoperative stone-free status, assessed using day-1 KUB and 1-month CT. The primary SFR endpoint was defined as no residual stones or a maximum residual fragment diameter of ≤4 mm, consistent with the EAU follow-up stratification for residual fragments measuring ≤4 mm and >4 mm. The strict 0 mm CT endpoint was defined as no residual fragments on 1-month CT, and it was analyzed as a sensitivity endpoint [1,8]. All included episodes underwent both day-1 KUB and postoperative CT assessment at approximately 1 month after surgery. Day-1 KUB was routinely obtained primarily to confirm D-J stent position and secondarily to provide an early assessment of residual radiopaque fragments; it was not used as a substitute for missing CT. CT was used as the reference imaging modality for residual-fragment assessment and, in patients with postoperative D-J stent placement, was performed after stent removal. When KUB and CT findings were discordant, endpoint classification was based on the CT finding.

The secondary outcome was operative time, defined as the interval from the start to end of surgery, in minutes. Operative time was analyzed in two ways. First, the whole cohort of 352 episodes was analyzed to assess the association between stone burden and overall operative duration. Second, an exploratory analysis was restricted to stone-free episodes, allowing for evaluation of operative time among procedures in which the postoperative stone-free endpoint was achieved.

### 2.3. Clinical and Operative Variables

Baseline and perioperative variables included age, sex, body mass index (BMI), estimated glomerular filtration rate (eGFR), chronic kidney disease (CKD) G stage or CKD G3+, diabetes mellitus, hypertension, coronary heart disease, ongoing antithrombotic therapy, history of malignancy, complex urological anatomy, preoperative double-J (D-J) stent, scope outer diameter, surgical position, stone laterality, lower-calyx involvement, and staghorn stones.

### 2.4. Operative Technique

All procedures were performed under general anesthesia by the same high-volume endourology team led by a senior surgeon with more than 30 years of experience in urolithiasis and extensive experience in flexible ureteroscopy. Flexible ureteroscopy was mainly performed using Zebra 8.6-Fr and Zebra 7.5-Fr ureteroscopes (Happiness Works Medical Instruments Co., Ltd., Bengbu, China), or Pusen 7.5-Fr ureteroscopes (Zhuhai Pusen Medical Technology Co., Ltd., Zhuhai, China), with Storz semirigid ureteroscopy (KARL STORZ SE & Co. KG, Tuttlingen, Germany), Boston Scientific or Cook guidewires (Boston Scientific Corporation, Marlborough, MA, USA; Cook Medical, Bloomington, IN, USA), and negative-pressure suction access sheaths used according to ureteral access and surgeon judgment. Suction access sheaths were procured from Zhejiang YiGao Medical Technology Co., Ltd. (Hangzhou, China); 40 cm and 50 cm sheaths were generally used in female and male patients, respectively, with 11 Fr sheaths used in most cases and 10 or 12 Fr sheaths used selectively. Lithotripsy was performed using a Lumenis Ho:YAG laser (Lumenis Ltd., Yokneam, Israel) with a 200 µm fiber, most commonly at 0.8 J and 25 Hz, with adjustment according to stone characteristics and intraoperative visibility. The usual strategy was fragmentation followed by suction-assisted evacuation of fragments and dust; basket extraction was used selectively, and lower-pole stone relocation was uncommon. The procedure was stopped after complete visible clearance or when further manipulation was considered unsuitable because of poor patient tolerance, poor visibility, bleeding, concerns regarding infection, difficult access, excessive operative durations, or residual burden better managed by staged treatment. In 38 synchronous bilateral procedures, operative time was recorded as the total duration of the same surgical episode. No major changes in the laser platform or suction-sheath strategy occurred during the study period.

### 2.5. CT Imaging Protocol and Stone-Level Measurements

Preoperative NCCT performed 1–15 days before surgery was used for all CT-derived measurements. All final included episodes had 5 mm routine reconstructions and 1 mm thin-section reconstructions; the 1 mm reconstructed images were used for stone-level measurements. Postoperative CT included both 5 mm routine reconstruction and 1 mm thin-section reconstruction. Residual fragments were assessed using the 1 mm thin-section images, with multiplanar reconstruction when needed. CT images were reviewed on an institutional picture-archiving and communication system (PACS) using multiplanar reconstruction when needed.

For each stone, measurements were performed on the plane showing the largest visible cross-sectional stone profile. The long diameter (L_i) was defined as the maximum caliper distance across that profile, and the short diameter (S_i) was defined as the largest diameter perpendicular to the long axis on the same profile. If a recorded long diameter was smaller than the short diameter during data checking, the two values were exchanged according to these definitions. For each stone with available attenuation data, Hounsfield units (HU) were measured on the largest cross-sectional profile using the default elliptical region-of-interest (ROI) tool. The ROI was placed within the stone to cover the largest feasible intrastone area while avoiding the peripheral edge, adjacent urine or soft tissue, beam-hardening artifacts, and partial-volume effects. This approach was used to reduce edge-related attenuation distortion, a recognized issue in CT-based stone density measurement [3,4,9].

Stone-level CT measurements were performed by one urologist. To assess interobserver reproducibility, a second urologist independently repeated the principal CT-derived stone measurements and burden-metric calculations in a random sample of 30 episodes, and intraclass correlation coefficients (ICCs) were calculated. All measurements and follow-up imaging assessments were reviewed by a senior urologist, and ambiguous stone boundaries, staghorn configuration, laterality, calyceal location, and postoperative residual-fragment assessment were adjudicated by consensus. Formal blinding to the preoperative burden metrics was not implemented because this was a retrospective review of routine clinical imaging; however, all image measurements and endpoint adjudications were completed before statistical modeling and were not modified after model development. Intraobserver repeatability was not separately evaluated.

### 2.6. CT-Derived Stone Burden Metrics

All stone burden metrics were calculated from stone-level records based on preoperative NCCT imaging. For each stone, long diameter, short diameter, location, and available CT attenuation were recorded. Stone burden metrics were calculated at the surgery-episode level according to the stones treated during that specific procedure. For patients with bilateral stones in whom only one side was treated during a given RIRS episode, only stones on the treated side were included in the burden calculation. In synchronous bilateral procedures, both sides were treated during the same surgical session, and the burden metrics were calculated by summing stones treated on both sides. The candidate metrics were grouped as follows:

1. Conventional one-dimensional burden metrics, including maximum stone diameter, CSD, and stone number;
2. Two-dimensional burden metrics, including estimated total area, area-equivalent diameter, area-weighted mean long diameter, and area-weighted mean short diameter;
3. Estimated three-dimensional burden metrics, including estimated total volume, volume-equivalent diameter, estimated total surface area, volume-weighted mean long diameter, and volume-weighted mean short diameter;
4. Morphological and distribution-complexity metrics, including aspect ratio, surface/volume ratio, dominant-stone area fraction, dominant-stone volume fraction, and volume distribution entropy;
5. Spatial distribution metrics, including lower-calyx involvement, multisite stones, stone site count, bilateral stones, and staghorn stones;
6. CT attenuation metrics, including mean stone attenuation, maximum stone attenuation, area-weighted mean stone attenuation, and volume-weighted mean stone attenuation.

Details regarding the formulas, units, and variable definitions are provided in Table S1.

Area-equivalent diameter was used to convert the total two-dimensional stone area into an intuitive single-diameter measure. Volume-equivalent diameter was used to convert estimated total stone volume into the diameter of a sphere with the same volume. Stone volume distribution entropy was used to describe how stone volume was distributed across multiple stones. We intended to use it to capture distribution complexity rather than total burden.

### 2.7. Training and Validation Sets

The final cohort of 352 episodes was randomly divided into a training set and a validation set in a 7:3 ratio using a fixed random seed of 800, with stratification by the primary SFR endpoint. The training set included 247 episodes, with 148 stone-free and 99 non-stone-free episodes. The validation set included 105 episodes, with 63 stone-free and 42 non-stone-free episodes. The baseline balance between the training and validation sets is shown in Table S2.

### 2.8. Statistical Analysis

All analyses were performed using R Foundation for Statistical Computing (R 4.5.2, Vienna, Austria). Continuous variables were summarized as means ± standard deviations or medians with interquartile ranges, as appropriate. Categorical variables were summarized as counts and percentages. Between-group comparisons were performed using tests appropriate for the types and distribution of the variables. There were complete data for all variables included in the present analyses, including CT-derived stone burden metrics, attenuation variables, operative time, and SFR endpoints. No imputation was performed. For SFR prediction, variable screening and model development were performed using the training set, with internal validation via the validation set. Univariable logistic regression was performed for candidate predictors. CT-derived stone burden metrics were then grouped using Spearman’s absolute-correlation clustering. Variables with |ρ| ≥ 0.85 were considered highly correlated. Within each cluster, up to two variables with the best univariable performance and *p* < 0.05 were retained for the multivariable candidate pool. Stepwise logistic regression was subsequently used to build the final model, retaining variables with *p* < 0.1 and clear clinical interpretability.

To assess the practical value of refined CT-derived metrics relative to conventional clinical assessment, a CSD model was also constructed. Both the main and CSD models were developed using the training set. Receiver operating characteristic (ROC) curves, the area under the curve (AUC), and Brier scores were calculated using both the training and validation sets. AUC and Brier scores were reported with 95% confidence intervals. Calibration was assessed using calibration curves, calibration intercepts, and calibration slopes. Decision curve analysis (DCA) was used to evaluate the clinical net benefit across threshold probabilities. For clinical interpretation, threshold probabilities of 0.30–0.80 were emphasized. Net-benefit gain was defined as the absolute increase in model net benefit over the better default strategy, namely, treat-all or treat-none, whichever yielded the higher net benefit at that threshold. Validation-set AUCs were compared using the DeLong test. Exploratory cutoffs for representative stone burden metrics were derived from the full analysis cohort using the Youden index and were not independently validated. To account for repeated episodes pertaining to the same patient, generalized estimating equations (GEEs) were fitted with patient ID as the clustering variable. In addition, 500 repeated patient-level 7:3 training–validation splits were performed. In each repeated split, all episodes from the same patient were assigned to the same split, and the full model-development pipeline was repeated within the training resample, including univariable screening, Spearman’s absolute-correlation clustering, cluster-wise candidate retention, and stepwise logistic regression. Model performance was then assessed in the corresponding validation resample. As a penalized-regression sensitivity analysis, LASSO logistic regression was performed using the candidate variables retained after correlation-cluster reduction. The penalty parameter was selected by 10-fold cross-validation in the training set using a fixed random seed of 800. To evaluate selection stability, 500 bootstrap resamples of the training set were generated, and variable-selection frequencies were calculated.

Operative time was analyzed using univariable linear regression and Spearman’s correlation. For each metric, the beta coefficient, 95% confidence interval (CI), *p* value, Spearman’s rho, training-set R^2^, validation-set R^2^, training-set root mean squared error (RMSE), validation-set RMSE, and validation-set mean absolute error (MAE) were calculated in the whole cohort and among stone-free episodes. For operative-time models, linearity, residual distribution, influential observations, and heteroscedasticity were assessed using fitted-value and residual diagnostics, Q-Q plots, Cook’s distance, and tests for heteroscedasticity. Additionally, log-transformed operative-time sensitivity analyses were performed to assess the robustness of the operative-time findings.

## 3. Results

### 3.1. Cohort Composition and Baseline Characteristics

Among the 485 screened RIRS episodes, 352 episodes pertaining to 322 unique patients were included in the final analysis. Of these 322 patients, 30 contributed two RIRS episodes during the study period. The primary SFR endpoint was achieved in 211 episodes (59.9%), whereas 141 episodes (40.1%) were not stone-free. The training and validation sets included 247 and 105 episodes, respectively. The study flow chart and analysis pathway are shown in Figure 1A. A comparison of the included and excluded RIRS episodes is provided in Table S3. Interobserver reproducibility was excellent for the principal CT-derived stone measurements and burden metrics, with ICCs ranging from 0.969 to 0.998 in a random sample of 30 episodes (Table S4).

In the overall cohort, 230 episodes (65.34%) involved male patients, and 122 (34.66%) involved female patients. The median BMI was 25.25 kg/m^2^. The median CSD was 24.95 mm overall, 19.60 mm in the stone-free group, and 36.30 mm in the non-stone-free group (*p* < 0.001). The median area-equivalent diameter was 13.51 mm overall, 12.13 mm in the stone-free group, and 17.83 mm in the non-stone-free group (*p* < 0.001). Stone number and stone volume distribution entropy were also higher in the non-stone-free group (both *p* < 0.001). Mean stone attenuation had a median of 1004.5 HU (IQR, 914.8–1063.0; range, 295.3–1632.0), and maximum stone attenuation had a median of 1065 HU (IQR, 1003.0–1208.5; range, 448–2242). Detailed baseline characteristics are presented in Table 1.

### 3.2. Univariable Analysis and Correlation Structure of Stone Burden Metrics

In univariable logistic regression, most stone burden metrics were significantly associated with the SFR. Among burden-size metrics, area-equivalent diameter (per 10 mm—odds ratio [OR], 0.170; 95% CI, 0.099–0.293; *p* < 0.001), volume-equivalent diameter (per 10 mm—OR, 0.116; 95% CI, 0.058–0.230; *p* < 0.001), CSD (per 10 mm—OR, 0.655; 95% CI, 0.563–0.763; *p* < 0.001), and estimated total surface area (per 100 mm^2^; OR, 0.836; 95% CI, 0.787–0.887; *p* < 0.001) were associated with lower SFRs. Among complexity and anatomical metrics, staghorn stones (OR, 0.149; 95% CI, 0.067–0.331; *p* < 0.001), lower-calyx involvement (OR, 0.326; 95% CI, 0.158–0.672; *p* = 0.001), stone volume distribution entropy (OR, 0.454; 95% CI, 0.287–0.719; *p* < 0.001), and stone number (per unit; OR, 0.863; 95% CI, 0.786–0.947; *p* = 0.002) were also associated with SFRs.

CT-derived stone burden metrics were clustered using Spearman’s absolute-correlation clustering, with variables showing |ρ| ≥ 0.85 considered highly correlated. After up to two variables per cluster with the best univariable performance and *p* < 0.05 were retained, the multivariable candidate pool included CSD, area-equivalent diameter, length-based area-equivalent diameter, stone number, stone volume distribution entropy, dominant-stone area and diameter fractions, largest-stone diameter descriptors, maximum stone attenuation, stone site count, lower-calyx involvement, multiple stones, and staghorn stones. The clustering pattern shows that area-, volume-, length-, and surface-area-based measures represented a highly correlated burden dimension, whereas volume distribution entropy and dominant-stone fraction captured distributional information not fully described by burden size alone (Figure 1B,C). The complete univariable logistic regression results and full cluster-wise reduction table are provided in Table S5 and Table S6, respectively, and the full Spearman’s correlation heatmap is shown in Figure S2.

### 3.3. Main Model for SFR

**Figure 2:**
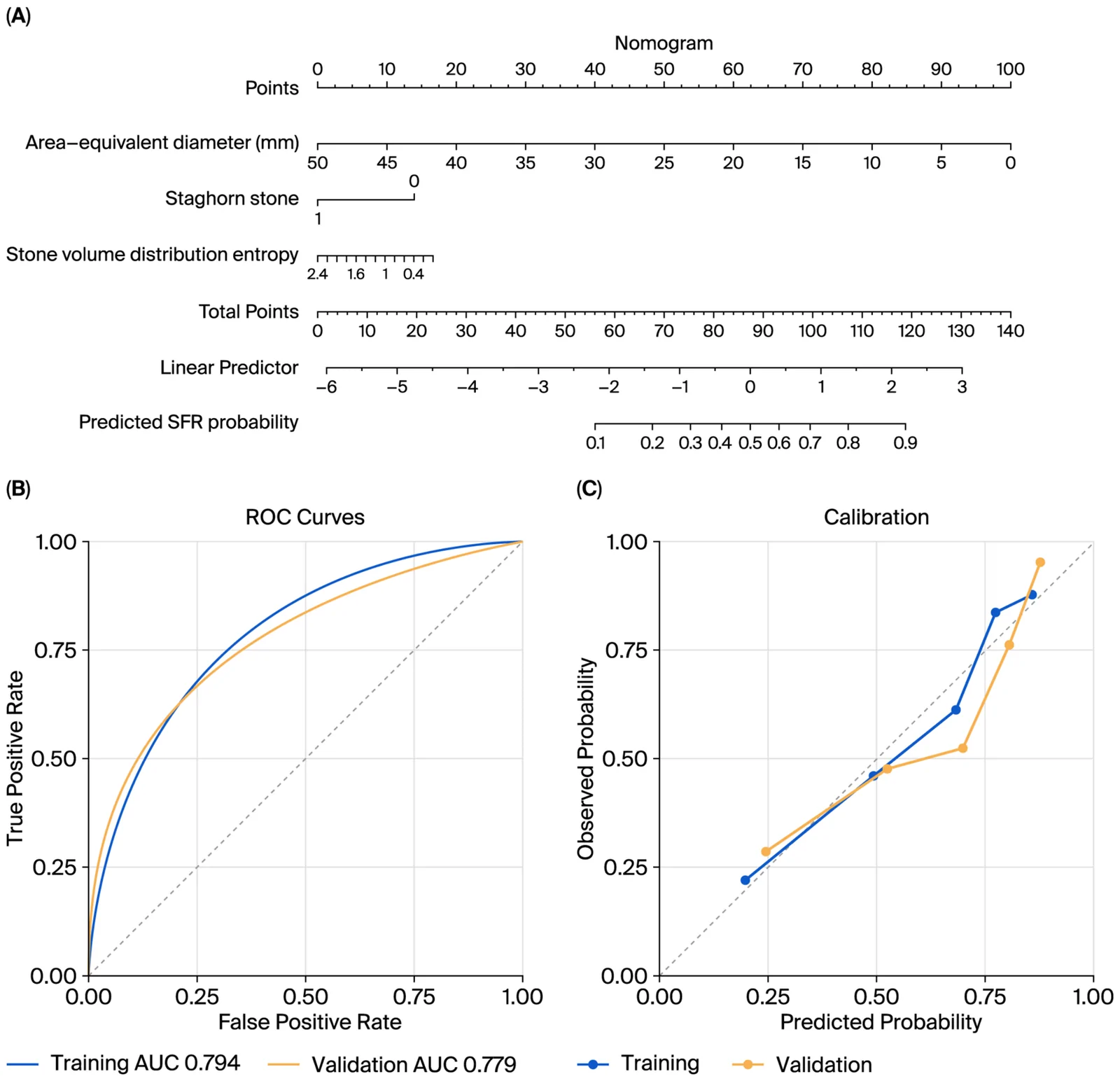

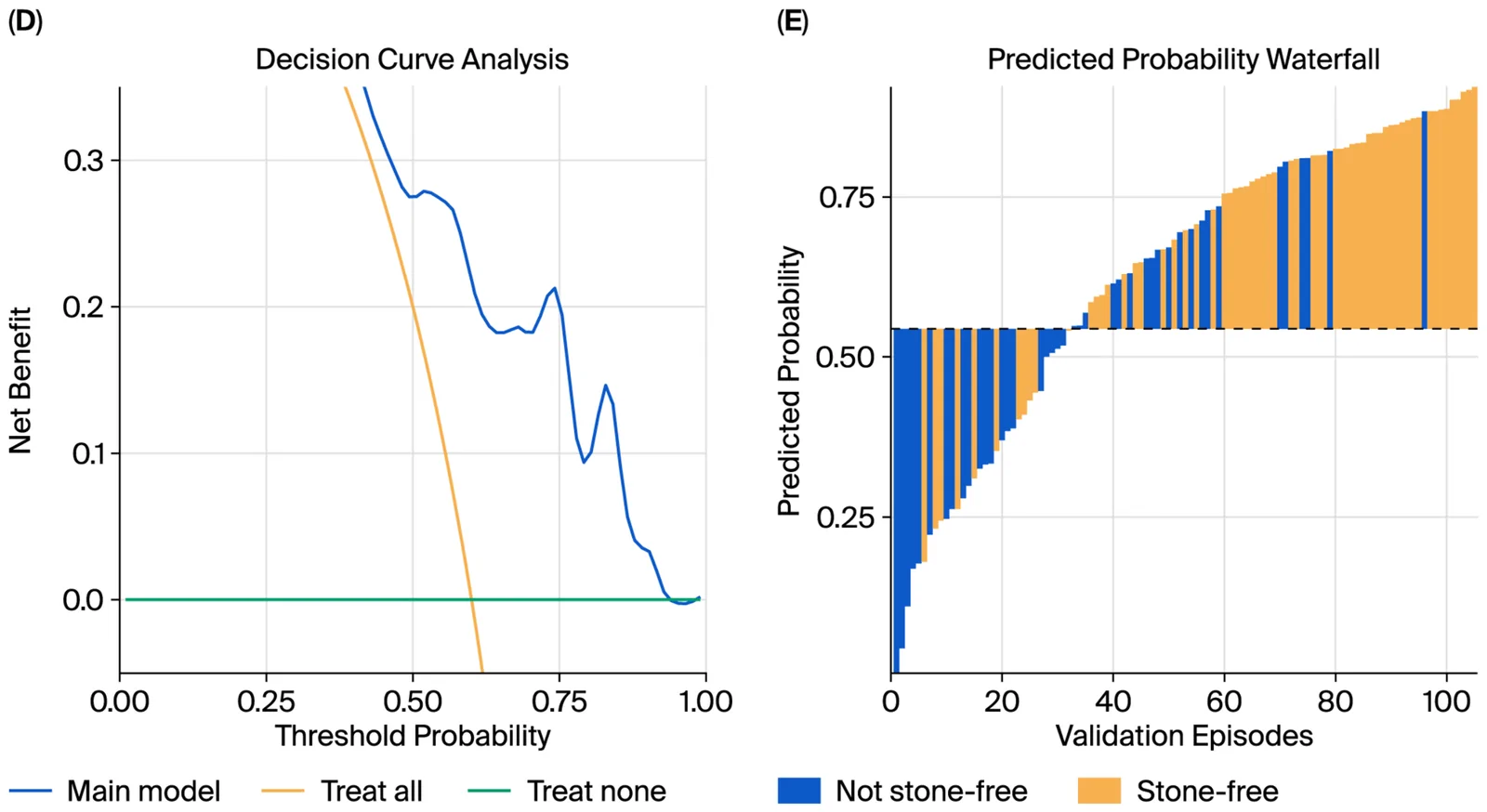
Main model for predicting stone-free status after RIRS: (**A**) nomogram; (**B**) receiver operating characteristic curves; (**C**) calibration curves; (**D**) clinical decision curve analysis; and (**E**) waterfall plot of validation-set predicted probabilities centered at the optimal cutoff. Dashed lines indicate the no-discrimination reference line in panel (**B**), the ideal calibration line in panel (**C**), and the selected probability cutoff in panel (**E**). ROC, receiver operating characteristic; AUC, area under the curve.

After univariable screening, correlation clustering, and stepwise logistic regression, the final main model included area-equivalent diameter, staghorn stones, and stone volume distribution entropy. A larger area-equivalent diameter was independently associated with a lower probability of stone-free status (OR, 0.869; 95% CI, 0.818–0.924; *p* < 0.001). Staghorn stones (OR, 0.377; 95% CI, 0.140–1.010; *p* = 0.052) and stone volume distribution entropy (OR, 0.615; 95% CI, 0.357–1.060; *p* = 0.080) showed consistent unfavorable directions (Figure 2A).

The main model achieved an AUC of 0.794 (95% CI, 0.737–0.851) and a Brier score of 0.180 (95% CI: 0.155–0.206) in regard to the training set. In the validation set, the AUC was 0.779 (95% CI: 0.690–0.867), and the Brier score was 0.194 (95% CI: 0.155–0.234) (Figure 2B and Table S7). Calibration curves showed acceptable agreement between predicted probabilities and observed SFRs, with a validation calibration intercept of −0.128 and slope of 0.932 (Figure 2C). In validation-set decision curve analysis, the main model provided a maximum absolute net-benefit gain of 0.218 at a threshold probability of 0.74, corresponding to 21.8 percentage points over the better default strategy (Figure 2D). In the validation set, the waterfall plot showed that stone-free episodes were mainly distributed above the optimal cutoff, whereas non-stone-free episodes were mainly distributed below it, indicating clinically interpretable individual-level risk stratification (Figure 2E). The full model equation, including the intercept, is provided in Table S7.

### 3.4. CSD Model

Given the practical convenience of CSD, a CSD model was constructed. The final CSD model included CSD and staghorn stones. CSD was negatively associated with stone-free status (OR, 0.964; 95% CI, 0.949–0.979; *p* < 0.001), as were staghorn stones (OR, 0.231; 95% CI, 0.099–0.538; *p* < 0.001).

The CSD model achieved a training-set AUC of 0.782 (95% CI, 0.724–0.840) and a Brier score of 0.189 (95% CI: 0.165–0.214). In the validation set, the AUC was 0.761 (95% CI: 0.670–0.852) and the Brier score was 0.203 (95% CI: 0.164–0.242). Calibration assessment showed a validation calibration intercept of −0.060 and a slope of 0.903 (Table S7). In validation-set decision curve analysis, the CSD model provided a maximum absolute net-benefit gain of 0.190 at a threshold probability of 0.78, corresponding to 19.0 percentage points over the better default strategy. Although the validation-set AUC was numerically higher for the main model than for the CSD model (0.779 vs. 0.761), the difference was not statistically significant according to the DeLong test (*p* = 0.201). NRI and IDI were retained as supportive measures: the validation-set continuous NRI was 0.460 (95% CI, 0.095–0.818; *p* = 0.007), and the IDI was 0.038 (95% CI, 0.011–0.068; *p* = 0.003). Thus, the area-equivalent diameter model offered a more refined expression of stone burden, whereas the CSD model retained strong practicality and interpretability. The model variables are shown in Table 2, and full equations and performance metrics are provided in Table S7. The CSD model nomogram and internal validation results are shown in Figure S1.

### 3.5. Sensitivity Analysis Using a Strict 0 mm CT Endpoint

A strict 0 mm CT endpoint was evaluated as a sensitivity analysis. Under this definition, 133/352 episodes (37.8%) were classified as strictly stone-free. When the original main-model variables were refitted for the strict 0 mm endpoint, the validation AUC was 0.670 (95% CI: 0.559–0.769), and the validation Brier score was 0.231 (95% CI: 0.191–0.274). The corresponding CSD model exhibited a validation AUC of 0.667 (95% CI: 0.554–0.767) and a validation Brier score of 0.233 (95% CI: 0.198–0.275). Model discrimination was lower under the strict endpoint, while the relative performance of the main model and the CSD model remained similar.

### 3.6. Sensitivity Analyses Accounting for Repeated Patients

Because 30 patients contributed two RIRS episodes during the study period, additional analyses were performed to assess the effect of patient-level clustering. In GEE models using patient ID as the clustering variable, the main model showed generally stable predictor effects for the primary SFR endpoint. Area-equivalent diameter remained associated with SFRs (OR, 0.256 per 10 mm; *p* < 0.001), while staghorn stones (OR, 0.367; *p* = 0.051) and stone volume distribution entropy (OR, 0.604; *p* = 0.069) remained directionally consistent. In the CSD model, CSD (OR, 0.695 per 10 mm; *p* < 0.001) and staghorn stones (OR, 0.219; *p* < 0.001) remained independently associated with SFRs.

To further evaluate the potential influence of episode-level splitting and model-selection instability, 500 repeated patient-level 7:3 training-validation splits were performed with the full model-development pipeline repeated within each training resample. All 500 repeated splits were successful. The median validation AUC of the repeated full-pipeline models was 0.761 (IQR, 0.738–0.787), and the median validation Brier score was 0.195 (IQR, 0.184–0.205). In the same patient-level splits, the fixed final main model showed a median validation AUC of 0.782 (IQR, 0.758–0.805) and a median Brier score of 0.187 (IQR, 0.177–0.197), whereas the fixed final CSD model showed a median validation AUC of 0.777 (IQR, 0.749–0.800) and a median Brier score of 0.195 (IQR, 0.185–0.204). These findings indicate that patient-level resampling yielded broadly similar performance estimates for the fixed final models, whereas repeated full-pipeline development produced a more conservative estimate (Table S8).

### 3.7. Incremental-Value Sensitivity Analysis Under Matched Covariates

In a matched-covariate sensitivity analysis, the CSD plus staghorn-stone model had a validation AUC of 0.761 and a validation Brier score of 0.203. Adding area-equivalent diameter to this model did not improve validation discrimination, with a validation AUC of 0.758 and a validation Brier score of 0.199, suggesting substantial overlap between CSD and area-equivalent diameter as burden-size descriptors. When stone volume distribution entropy was further added, the validation AUC was 0.783 and the validation Brier score was 0.189. These findings indicate that area-equivalent diameter alone did not provide clear incremental discrimination over CSD after accounting for staghorn morphology, whereas distributional information may provide supportive refinement.

### 3.8. Sensitivity Analysis Restricted to Unilateral Episodes

A unilateral-only sensitivity analysis was performed to assess whether synchronous bilateral procedures influenced the SFR prediction results. After excluding 38 synchronous bilateral episodes, 314 unilateral episodes remained. In this subset, the main model had a validation AUC of 0.768 and a validation Brier score of 0.194, whereas the CSD model had a validation AUC of 0.747 and a validation Brier score of 0.205. The relative performance of the two models was therefore similar to that observed in the primary cohort.

### 3.9. Penalized-Regression Sensitivity Analysis

As a penalized-regression sensitivity analysis, LASSO logistic regression was performed using the 14 variables retained after correlation-cluster reduction. At lambda.min, the model selected area-equivalent diameter, largest-volume stone diameter, maximum stone attenuation, stone site count, and staghorn stone, with a validation AUC of 0.760 and a validation Brier score of 0.198. At the more parsimonious lambda.1se, only area-equivalent diameter was retained, with a validation AUC of 0.755 and a validation Brier score of 0.233.

In 500 bootstrap resamples, area-equivalent diameter and staghorn stone showed high selection frequencies at lambda.min of 97.8% and 87.4%, respectively. Stone volume distribution entropy was selected in 37.4% of bootstrap resamples. In the repeated patient-level full-pipeline analysis, entropy was selected in 60.4% of successful splits. These findings support the stability of area-equivalent diameter and staghorn stone under the penalized-regression sensitivity framework while indicating that the incremental contribution of entropy was less stable and should be interpreted as supportive rather than definitive (Tables S8 and S9).

### 3.10. Operative Time Analysis for the Whole Cohort

In the whole-cohort operative time analysis, two-dimensional and estimated three-dimensional burden metrics showed stronger associations than CSD. Volume-equivalent diameter had a Spearman’s rho of 0.678, a training-set R^2^ of 0.376, and a validation-set R^2^ of 0.439. Area-equivalent diameter had a Spearman’s rho of 0.669, a training-set R^2^ of 0.358, and a validation-set R^2^ of 0.404. The validation-set R^2^ was 0.373 for maximum stone diameter, 0.298 for estimated total surface area, and 0.167 for CSD. Staghorn stones were also associated with longer operations. Representative results are shown in Table 3, and the complete results are provided in Table S10.

**Figure 3:**
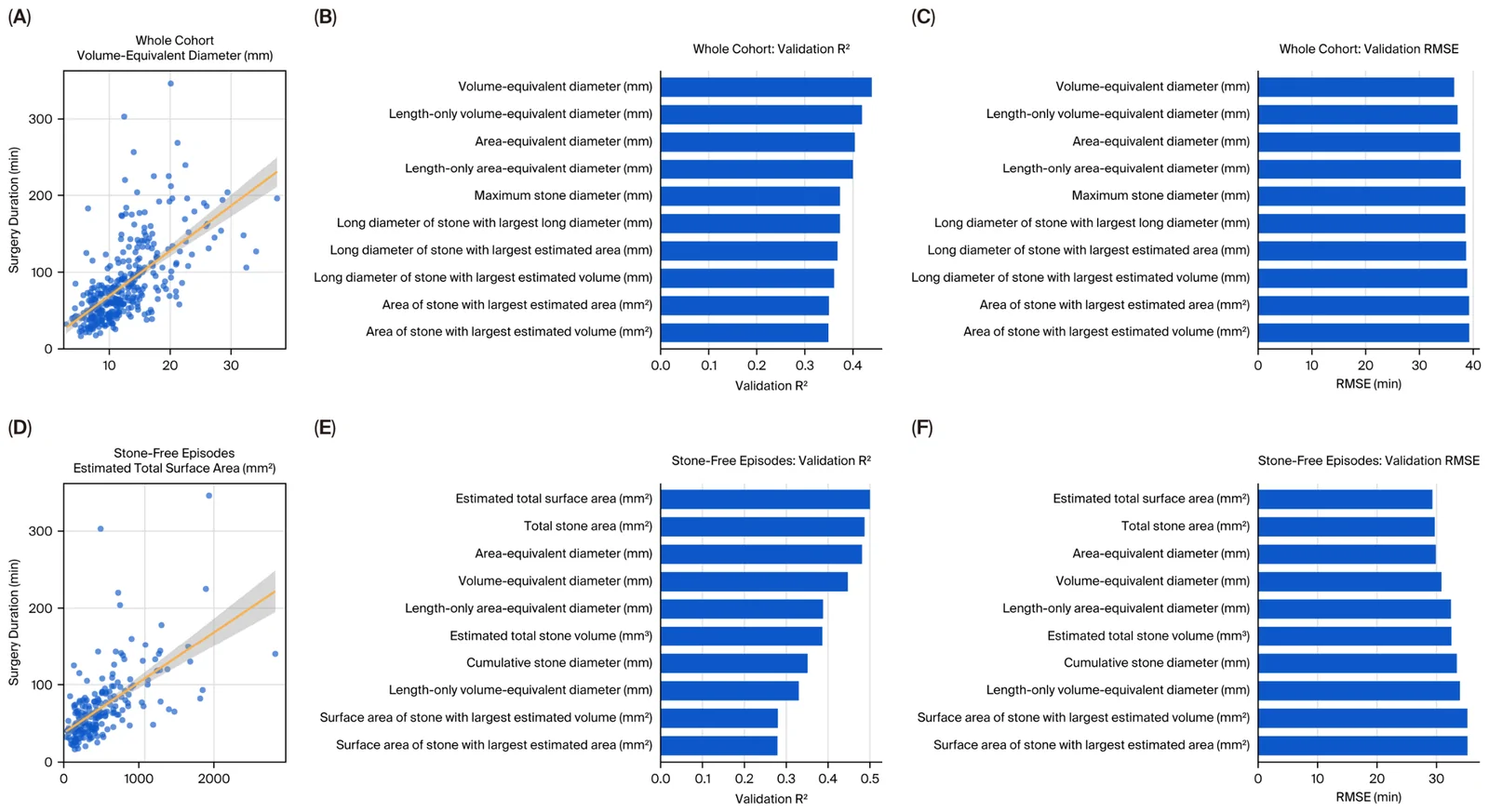
CT-derived stone burden metrics and operative time: (**A**) best-performing predictor in the whole cohort; (**B**) validation R^2^ for the whole cohort; (**C**) validation RMSE for the whole cohort; (**D**) best-performing predictor in stone-free episodes; (**E**) validation R^2^ for stone-free episodes; and (**F**) validation RMSE for stone-free episodes. In panels (**A**,**D**), the orange lines indicate fitted linear regression lines, and the gray bands indicate 95% confidence intervals.

These findings suggest that operative time reflects the practical workload of fragmentation and clearance more closely than CSD alone. This workload is shaped not only by summed long diameters but also by area, volume, and morphological complexity (Figure 3A–C). Diagnostic assessment of the raw-scale linear models revealed non-normal residuals and partial evidence of nonlinearity and heteroscedasticity. Cook’s distance identified isolated influential observations in a small number of raw-scale univariable models, but the main continuous burden-metric associations were not driven by a single observation. Therefore, log-transformed operative-time sensitivity analyses were performed.

### 3.11. Operative Time Analysis for Stone-Free Episodes

Among stone-free episodes, area-, volume-, and surface-area-based metrics remained strongly associated with operative time. Estimated total surface area had a Spearman’s rho of 0.655, a training-set R^2^ of 0.325, and a validation-set R^2^ of 0.500. Area-equivalent diameter had a Spearman’s rho of 0.653, a training-set R^2^ of 0.328, and a validation-set R^2^ of 0.481. Volume-equivalent diameter had a Spearman’s rho of 0.653, a training-set R^2^ of 0.329, and a validation-set R^2^ of 0.447. CSD remained significant in this subgroup, but its validation-set R^2^ was 0.351, lower than those of the above two-dimensional and three-dimensional metrics (Figure 3D–F and Figure S3, Table 3 and Table S11). Log-transformed operative-time sensitivity analyses were performed to assess the robustness of the raw-scale findings. After log transformation, residual normality improved in 45/45 whole-cohort models and 42/45 stone-free-episode models. No representative area- or volume-equivalent diameter model had Cook’s distance > 1. In the whole cohort, volume-equivalent diameter showed the highest validation R^2^ among representative metrics (0.445), followed by area-equivalent diameter (0.415), whereas CSD showed a lower validation R^2^ (0.180). Among stone-free episodes, area-equivalent diameter showed the highest validation R^2^ (0.464), followed by volume-equivalent diameter (0.448), whereas CSD again showed a lower validation R^2^ (0.297) (Table S12). These findings support the robustness of the operative-time results after log transformation, while the stone-free subgroup analysis should be interpreted as exploratory.

## 4. Discussion

Preoperative NCCT carried useful information for both major endpoints, but the useful information was not identical for SFRs and operative time. For the SFR, the final model retained area-equivalent diameter, staghorn stones, and stone volume distribution entropy. A CSD-plus-staghorn model achieved a similar AUC, which is important because it can be measured rapidly in ordinary clinical work. Reclassification, however, favored the model with area-equivalent diameter and entropy. For operative time, the contrast was sharper: area-, volume-, and surface-area-based metrics tracked the duration of surgery better than CSD.

This split is clinically plausible. The SFR is a thresholded outcome: a case is either classified as stone-free or not, using a defined residual-fragment cutoff. In this setting, CSD captures much of the gross burden once a staghorn morphology has been identified [10]. Operative time is different. It accumulates in every step of stone treatment, from reaching each calyx to fragmenting, inspecting, relocating, and clearing the fragments. A two- or three-dimensional burden estimate is naturally closer to this workload [11].

The broader direction is consistent with recent systematic evidence favoring multidimensional assessment of stone burden [4]. It also reflects a change in how endourologists think about size: an international survey showed that urologists increasingly regard stone volume as more informative than maximum diameter [12]. Our data add practical qualification to this view. Volume- and area-related metrics are not simply more elegant measurements; they appear to describe operative work better than linear size.

CSD should not be dismissed. Its strength is precisely that it is ordinary. It is fast, reproducible enough for clinics, and embedded in treatment selection [1,13]. In this cohort, it remained strongly associated with SFRs, and, when paired with staghorn morphology, it produced a useful clinical model [4]. This agrees with systematic evidence showing that multidimensional measures often add information, while linear measures remain widely used because of their simplicity [4]. For clinics lacking the capacity for structured stone-level CT extraction, CSD plus staghorn morphology is probably the most deployable model [14].

Area-equivalent diameter is a useful compromise between simplicity and geometry. It does not require the reader to think in square millimeters, but it still uses both the long and short axes of every stone. By converting total estimated area back into a diameter, it yields a number that is easy to interpret while retaining shape information that CSD loses. Earlier RIRS work and recent systematic evidence support the value of volume- or area-related burden metrics for predicting SFR and operative time [4,7,15]. In our cohort, the area-equivalent model had a numerically higher validation AUC than the CSD model, although the difference was not statistically significant. This finding makes the clinical message fairly balanced: area-equivalent diameter is preferable when stone-level CT data are already available, while CSD remains a reasonable rapid proxy.

The reclassification results point in the same direction. Compared with the CSD model, the main model improved the discrimination slope and reclassified patients more favorably, especially among stone-free episodes. In other words, the more detailed CT metrics mainly sharpened the estimate of who was likely to be cleared rather than replacing CSD as a broad marker of risk.

Stone volume distribution entropy deserves a short explanation because it is less familiar than diameter or area. It does not measure the amount of stone; it measures how the stone’s volume is divided. Consider two patients with an estimated total volume of 500 mm^3^. One has a 490 mm^3^ main stone and a 10 mm^3^ satellite stone. The other has five 100 mm^3^ stones in different calyces. The second patient has higher entropy. The burden is not larger, but it is more dispersed, and clearance usually requires more inspection and retrieval. The adverse direction of entropy in the main model suggests that this internal distribution of stones can affect the SFR. However, its selection stability was lower than that of area-equivalent diameter and staghorn morphology in the penalized-regression sensitivity analysis. Entropy should therefore be interpreted as a supportive distributional feature rather than a definitive independent predictor. Its practical limitation is also obvious: entropy requires reliable stone-level data.

The staghorn result necessitates little statistical persuasion. A staghorn stone brings together a large burden, a branching geometry, renal pelvic and calyceal involvement, and difficult clearance routes [14]. Existing RIRS scores also place size, number, location, and anatomical complexity near the center of SFR prediction [5,6]. Flexible ureteroscopy can be used in selected staghorn cases, especially with modern laser systems, but clearance remains constrained by the shape and extent of the stone, and staged treatment is often necessary [13]. Its retention in the final models is therefore clinically coherent.

Lower-calyx involvement was significant in univariable analysis (OR, 0.326; 95% CI, 0.158–0.672; *p* = 0.001), although it did not remain significant in the final multivariable model. This finding should not be interpreted as the absence of an effect. Real-world ureteroscopy data and earlier scoring systems repeatedly link lower-pole location, multiplicity, and stone size with residual fragments [6,16,17]. In this cohort, the lower-calyx signal was probably shared with total burden, staghorn morphology, and distribution complexity. It remains a useful feature to note before surgery, even if it was not an independent term in the final model.

CT attenuation contributed less than stone geometry. Maximum stone attenuation was associated with SFRs in univariable analysis (per 100 HU—OR, 0.909; 95% CI, 0.829–0.997; *p* = 0.044), and mean stone attenuation showed a borderline association (per 100 HU—OR, 0.907; 95% CI, 0.810–1.017; *p* = 0.095). Neither entered the final model. Stone density has been used in scoring and nomogram studies [6], and hardness plainly affects fragmentation. In this cohort, however, SFRs appeared to be driven more by how much stone was present, how it was shaped, and how it was distributed. This may partly reflect contemporary flexible ureteroscopy and laser lithotripsy, which can reduce the relative disadvantage of high-density stones. However, the relatively high and narrow attenuation distribution in this tertiary surgical cohort should also be considered. Restricted attenuation range may have reduced the apparent predictive value of HU-based variables.

Operative time provided the clearest support for multidimensional burden metrics [18]. The SFR is the more clinically direct endpoint, but it is binary and depends on the residual-fragment threshold, follow-up imaging, staging decisions, and safety judgment [19]. In the strict 0 mm CT sensitivity analysis, model discrimination was lower, with a validation AUC of 0.670 for the main model and 0.667 for the CSD model. This finding emphasizes that the primary ≤ 4 mm endpoint should not be equated with complete clearance. Operative time is noisier, but it records workload continuously [20]. Prior RIRS work identified stone volume as an important determinant of operative duration [7]. We saw the same pattern. Volume-equivalent diameter, area-equivalent diameter, and estimated total surface area explained operative time much better than CSD, especially among stone-free episodes. This is the setting in which two-dimensional and estimated three-dimensional CT metrics may have their most immediate use: estimating how much work the case will require [12]. However, these operative-time comparisons were primarily univariable and should be interpreted as descriptive associations rather than evidence of independent predictive contribution after adjustment for all operative and anatomical factors.

A practical interpretation of these findings is that it is prudent to use different metrics for different decisions. CSD plus staghorn morphology is enough for rapid counseling about SFRs in many clinics. If stone-level CT extraction is available, area-equivalent diameter and volume distribution entropy may refine the risk estimate, although the incremental advantage over CSD for binary SFR prediction was modest. For operative scheduling, room planning, and discussion of staged treatment, area-, volume-, and surface-area-based metrics appear more informative than CSD alone.

The cutoff analysis was exploratory, but the numbers are clinically recognizable. A 20 mm threshold has long shaped RIRS treatment selection and expectations for clearance [1,21]. In our study, the optimal CSD cutoff was 23.1 mm (AUC, 0.759; sensitivity, 0.787; specificity, 0.640; F1, 0.677), which is close to that boundary. CSD ≥ 20 mm favored sensitivity (0.844), whereas CSD ≥ 25 mm improved specificity (0.659) and yielded a more balanced profile. The shift toward 25 mm may reflect the use of modern RIRS instruments, access sheaths, laser platforms, and team experience, which make stones around 20 mm less prohibitive than in earlier eras [10,16]. For area-equivalent diameter, the optimal cutoff was 13.2 mm (AUC, 0.767; sensitivity, 0.787; specificity, 0.640; F1, 0.677); 10 mm favored sensitivity (0.894), while 15 mm favored specificity (0.735) and accuracy (0.705). Stone number was weaker alone but still easy to understand: the optimal cutoff was close to ≥5 (AUC, 0.630; sensitivity, 0.355; specificity, 0.853; F1, 0.450), with ≥4 yielding higher sensitivity (0.440) and ≥5 yielding higher specificity (0.853) (Table S13). This outcome agrees with recent nomogram work linking higher stone numbers to non-stone-free status [16] and with earlier RIRS scores that treated multiplicity as part of complexity [5]. Stone number is best used as a supplement, not as the central burden metric [22,23].

Several limitations should be considered. First, this study is a retrospective, episode-based analysis pertaining to a single high-volume surgical team, and 30 patients contributed two RIRS episodes. Although sensitivity analyses accounting for patient-level clustering showed generally stable results, repeated episodes and the single-team setting may limit generalizability. Second, the cohort was treated by an experienced lead surgeon; this fact reduced heterogeneity in operative technique but does not exclude the possibility of temporal changes in operative practices during the study period. Third, the area, volume, and surface area variables were estimated from long- and short-axis measurements rather than from true three-dimensional segmentation, and the former may be less precise for irregular or staghorn stones. Standardized three-dimensional segmentation, radiomics, or automated CT image analysis may provide more precise and reproducible burden estimates in future studies [24,25,26]. Fourth, although interobserver reproducibility was excellent, formal blinding to preoperative burden metrics and intraobserver repeatability assessment were not implemented. Fifth, some operative factors, including detailed calyceal anatomy, irrigation conditions, intrarenal pressure, laser energy delivery, stone composition, and intraoperative visibility, were not fully captured. Sixth, the operative-time analysis restricted to stone-free episodes was exploratory because it was conditioned on a postoperative outcome. Finally, external validation was not conducted; therefore, the models should be interpreted as internally validated development models rather than tools ready for immediate clinical implementation.

## 5. Conclusions

Preoperative CT-derived stone burden metrics showed potential for predicting stone-free status and operative time after RIRS in this single-center model-development and internal-validation study. For the primary SFR endpoint, area-equivalent diameter, staghorn stone, and stone volume distribution entropy formed the main model, while CSD plus staghorn stone offered a simpler model with comparable discrimination. The incremental value of entropy should be interpreted cautiously. For operative time, area-, volume-, and surface-area-based metrics were more informative than CSD. These findings suggest that multidimensional CT metrics may help refine preoperative assessments and operative planning, but external validation is required before clinical implementation.

## Figures and Tables

**Table 1:** Baseline characteristics according to stone-free status.

Variable	Overall	Stone-Free (*n* = 211)	Not Stone-Free (*n* = 141)	Statistic (t/Z/χ^2^)	*p*
Operative time (min)	70.0 [47.00, 106.00]	61.00 [42.00, 83.50]	90.00 [59.00, 137.00]	6.108	<0.001
Age (years)	54.37 (±12.37)	55.03 (±12.93)	53.38 (±11.43)	1.264	0.207
Sex				1.8	0.180
Female	122 (34.66)	79 (37.44)	43 (30.50)		
Male	230 (65.34)	132 (62.56)	98 (69.50)		
BMI (kg/m^2^)	25.25 [22.90, 27.34]	25.22 [22.80, 27.32]	25.25 [22.99, 27.34]	0.448	0.654
eGFR (mL/min/1.73 m^2^)	100.30 [86.20, 107.73]	100.40 [88.90, 106.65]	99.20 [83.10, 108.40]	0.531	0.596
Hyperuricemia or gout				0.22	0.639
No	252 (71.59)	153 (72.51)	99 (70.21)		
Yes	100 (28.41)	58 (27.49)	42 (29.79)		
Renal tubular acidosis				5.269	0.022
No	347 (98.58)	211 (100.00)	136 (96.45)		
Yes	5 (1.42)	0 (0.00)	5 (3.55)		
Sjogren syndrome				6.771	0.009
No	346 (98.30)	211 (100.00)	135 (95.74)		
Yes	6 (1.70)	0 (0.00)	6 (4.26)		
Complex urological anatomy				1.43	0.232
No	304 (86.36)	186 (88.15)	118 (83.69)		
Yes	48 (13.64)	25 (11.85)	23 (16.31)		
Scope outer diameter (Fr)	7.50 [7.50, 7.50]	7.50 [7.50, 7.50]	7.50 [7.50, 8.60]	2.223	0.003
Surgical position				7.007	0.136
Galdakao-modified supine Valdivia (GMSV)	4 (1.14)	1 (0.47)	3 (2.13)		
Left lateral decubitus	1 (0.28)	0 (0.00)	1 (0.71)		
Lithotomy	100 (28.41)	55 (26.07)	45 (31.91)		
Supine	1 (0.28)	0 (0.00)	1 (0.71)		
Supine split leg	246 (69.89)	155 (73.46)	91 (64.54)		
Stone laterality				1.6	0.449
Bilateral	38 (10.80)	20 (9.48)	18 (12.77)		
Left	190 (53.98)	119 (56.40)	71 (50.35)		
Right	124 (35.23)	72 (34.12)	52 (36.88)		
Maximum stone diameter (mm)	13.90 [10.15, 18.50]	12.60 [9.10, 15.85]	16.10 [11.90, 25.10]	5.752	<0.001
Cumulative stone diameter (mm)	24.95 [15.67, 39.62]	19.60 [13.60, 29.70]	36.30 [24.30, 52.30]	8.235	<0.001
Stone number	2.00 [1.00, 4.00]	2.00 [1.00, 4.00]	3.00 [2.00, 6.00]	4.129	<0.001
Area-equivalent diameter (mm)	13.51 [9.99, 18.87]	12.13 [8.89, 15.47]	17.83 [13.35, 22.73]	8.483	<0.001
Stone volume distribution entropy	0.48 [0.00, 0.93]	0.39 [0.00, 0.71]	0.61 [0.14, 1.19]	3.948	<0.001
Mean stone attenuation (HU)	1004.50 [914.75, 1063.00]	997.33 [889.33, 1060.67]	1014.50 [938.33, 1071.60]	1.18	0.238
Maximum stone attenuation (HU)	1065.00 [1003.00, 1208.50]	1059.00 [987.00, 1182.00]	1093.00 [1022.00, 1226.00]	1.983	0.047

**Table 2:** Final multivariate logistic regression models for stone-free status.

Model	Variable	OR (95% CI)	*p*
Main model	Intercept	20.620 (8.335–51.012)	<0.001
Main model	Area-equivalent diameter (mm)	0.869 (0.818–0.924)	<0.001
Main model	Staghorn stones	0.377 (0.140–1.010)	0.052
Main model	Stone volume distribution entropy	0.615 (0.357–1.060)	0.080
CSD model	Intercept	6.295 (3.579–11.073)	<0.001
CSD model	Cumulative stone diameter (mm)	0.964 (0.949–0.979)	<0.001
CSD model	Staghorn stones	0.231 (0.099–0.538)	<0.001

**Table 3:** Representative operative-time predictors.

Analysis	Variable	Beta (95% CI)	*p*	Spearman’s rho	Spearman’s *p*	Training R^2^	Validation R^2^	Training RMSE	Validation RMSE	Validation MAE
Whole cohort	Maximum stone diameter (mm)	3.599 (2.940–4.258)	<0.001	0.591	<0.001	0.319	0.373	42.745	38.540	28.241
Whole cohort	Cumulative stone diameter (mm)	0.893 (0.649–1.137)	<0.001	0.530	<0.001	0.174	0.167	47.075	44.411	34.204
Whole cohort	Area-equivalent diameter (mm)	4.284 (3.565–5.003)	<0.001	0.669	<0.001	0.358	0.404	41.508	37.559	27.597
Whole cohort	Volume-equivalent diameter (mm)	5.682 (4.765–6.600)	<0.001	0.678	<0.001	0.376	0.439	40.929	36.452	26.872
Whole cohort	Estimated total surface area (mm^2^)	0.031 (0.025–0.038)	<0.001	0.669	<0.001	0.283	0.298	43.861	40.756	31.103
Whole cohort	Estimated surface/volume ratio	−73.963 (−91.259–−56.667)	<0.001	−0.586	<0.001	0.223	0.290	45.659	40.996	31.975
Whole cohort	Staghorn stones	57.633 (41.377–73.889)	<0.001			0.165	0.143	47.338	45.052	34.994
Stone-free episodes	Maximum stone diameter (mm)	3.503 (2.149–4.856)	<0.001	0.495	<0.001	0.150	0.226	41.780	36.491	25.104
Stone-free episodes	Cumulative stone diameter (mm)	1.165 (0.785–1.545)	<0.001	0.528	<0.001	0.198	0.351	40.571	33.412	24.277
Stone-free episodes	Area-equivalent diameter (mm)	5.336 (4.096–6.576)	<0.001	0.653	<0.001	0.328	0.481	37.155	29.882	21.546
Stone-free episodes	Volume-equivalent diameter (mm)	7.141 (5.487–8.795)	<0.001	0.653	<0.001	0.329	0.447	37.111	30.845	22.530
Stone-free episodes	Estimated total surface area (mm^2^)	0.061 (0.047–0.075)	<0.001	0.655	<0.001	0.325	0.500	37.216	29.308	20.811
Stone-free episodes	Estimated surface/volume ratio	−53.014 (−74.224–−31.805)	<0.001	−0.513	<0.001	0.141	0.206	41.990	36.944	27.757
Stone-free episodes	Stone volume distribution entropy	20.270 (5.975–34.565)	0.006	0.254	0.002	0.050	0.080	44.157	39.780	30.170
Stone-free episodes	Stone number	3.397 (0.658–6.136)	0.016	0.271	<0.001	0.039	0.050	44.420	40.410	30.835
Stone-free episodes	Staghorn stones	49.929 (20.260–79.598)	0.001			0.069	0.078	43.711	39.819	30.012

## Data Availability

The original contributions presented in this study are included in the article/Supplementary Material. Further inquiries can be directed to the corresponding authors.

## Supplementary Materials

The following supporting information can be downloaded at https://www.mdpi.com/article/10.3390/diagnostics16182994/s1. Figure S1: CSD-based model for predicting stone-free status after RIRS. Figure S2: Correlation structure of CT-derived stone burden metrics: full Spearman’s correlation heatmap of candidate stone burden variables. Figure S3: Operative-time prediction via CT-derived stone burden metrics. Table S1: Definitions and formulas of CT-derived stone burden metrics. Table S2: Baseline characteristics of the training and validation sets. Table S3: Comparison of included and excluded RIRS episodes. Table S4: Interobserver reproducibility of CT-derived stone measurements and burden metrics. Table S5: Complete univariable logistic regression analysis for stone-free status. Table S6: Spearman’s correlation clustering and retained candidate variables for stone-free status prediction. Table S7: Full model equations, AUC values, and Brier scores with 95% CIs, calibration intercepts, and slopes. Table S8: Repeated patient-level full-pipeline validation for stone-free status prediction. Table S9: Penalized-regression sensitivity analysis and bootstrap selection frequencies. Table S10: Complete univariable linear regression analysis for operative time in the whole cohort. Table S11: Complete univariable linear regression analysis for operative time among stone-free episodes. Table S12: Log-transformed operative-time sensitivity analysis. Table S13: Exploratory cutoff performance of representative stone burden metrics in the full cohort.

## Abbreviations

The following abbreviations are used in this manuscript:

AUC	area under the receiver operating characteristic curve
BMI	body mass index
CI	confidence interval
CKD	chronic kidney disease
CSD	cumulative stone diameter
CT	computed tomography
DCA	decision curve analysis
D-J	double-J
EAU	European Association of Urology
eGFR	estimated glomerular filtration rate
GEE	generalized estimating equations
HU	Hounsfield unit
ICC	intraclass correlation coefficient
IDI	integrated discrimination improvement
KUB	kidney–ureter–bladder radiography
MAE	mean absolute error
NCCT	non-contrast computed tomography
NPV	negative predictive value
NRI	net reclassification improvement
OR	odds ratio
PACS	picture-archiving and communication system
PPV	positive predictive value
RIRS	retrograde intrarenal surgery
ROC	receiver operating characteristic
ROI	region of interest
RMSE	root mean squared error
SD	standard deviation
SFR	stone-free rate
