# Supplementary material for: Preoperative CT-Derived Stone Burden Metrics for Predicting Stone-Free Status and Operative Time After Retrograde Intrarenal Surgery
Source: Diagnostics (Basel). 2026 Sep 16;16(18):2994. doi: 10.3390/diagnostics16182994 (PMC13605811; doi:10.3390/diagnostics16182994)
Supplement: Supplementary file 1 [file diagnostics-16-02994-s001.zip › diagnostics-4513291-supplementary.pdf]

Supplementary Materials for

Preoperative CT-derived stone burden metrics for predicting stone-free status and operative time after retrograde intrarenal surgery

Shiwei Sun, Jiang Liu, Yi Qiao, Dong Wang, Hongjun Li, and He Xiao

This file includes:

Supplementary Figures S1 to S3

Supplementary Tables S1 to S13

Supplementary Figures

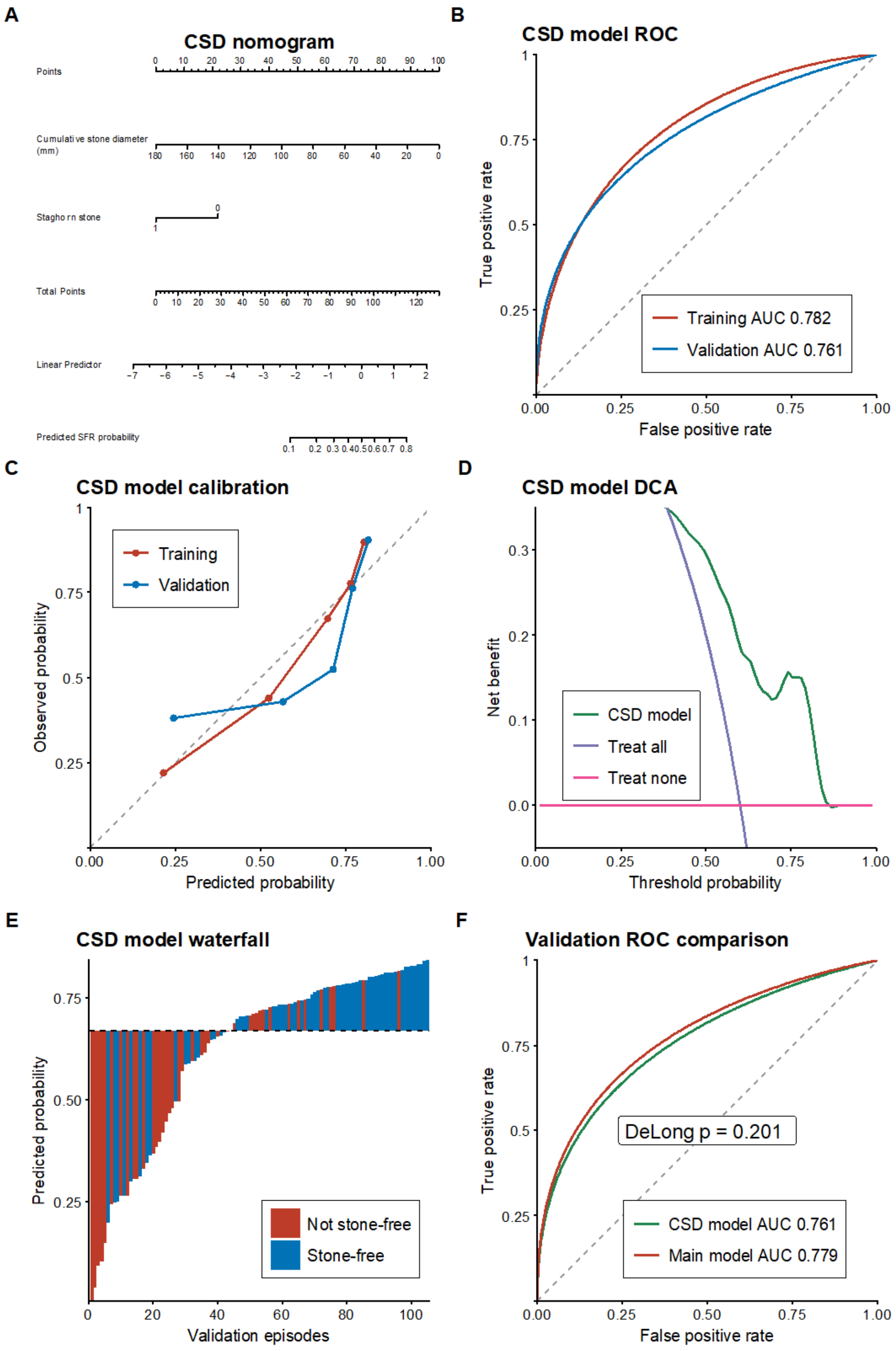

Figure S1. CSD-based model for predicting stone-free status after RIRS.

(A) Nomogram; (B) Receiver operating characteristic curves; (C) Calibration curves; (D) Clinical decision curve analysis; (E) Waterfall plot of validation-set predicted probabilities centered at the optimal cutoff; (F) Receiver operating characteristic curve comparison between the main model and the CSD-based model.

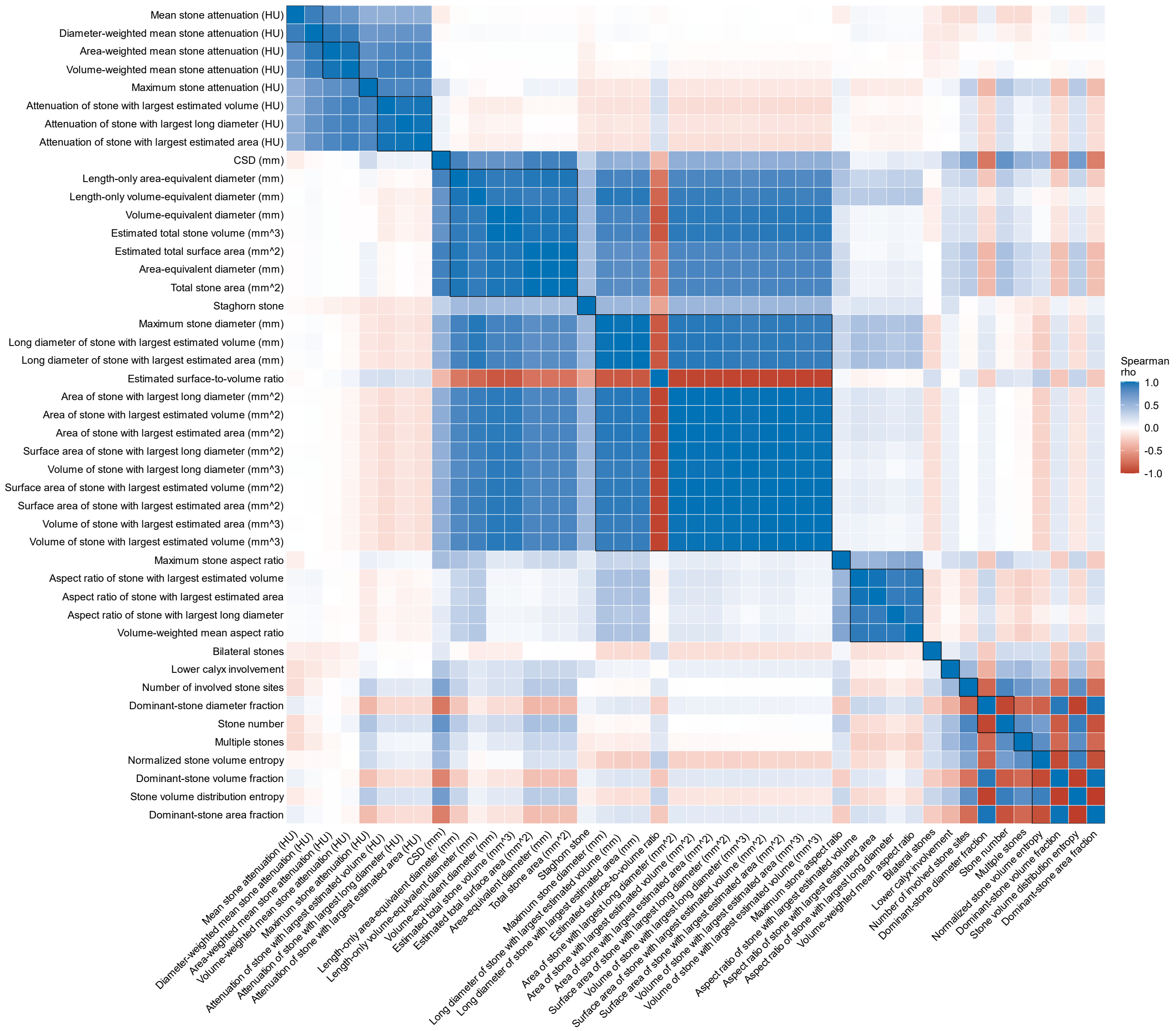

Figure S2. Correlation structure of CT-derived stone burden metrics: full Spearman's correlation heatmap of candidate stone burden variables.

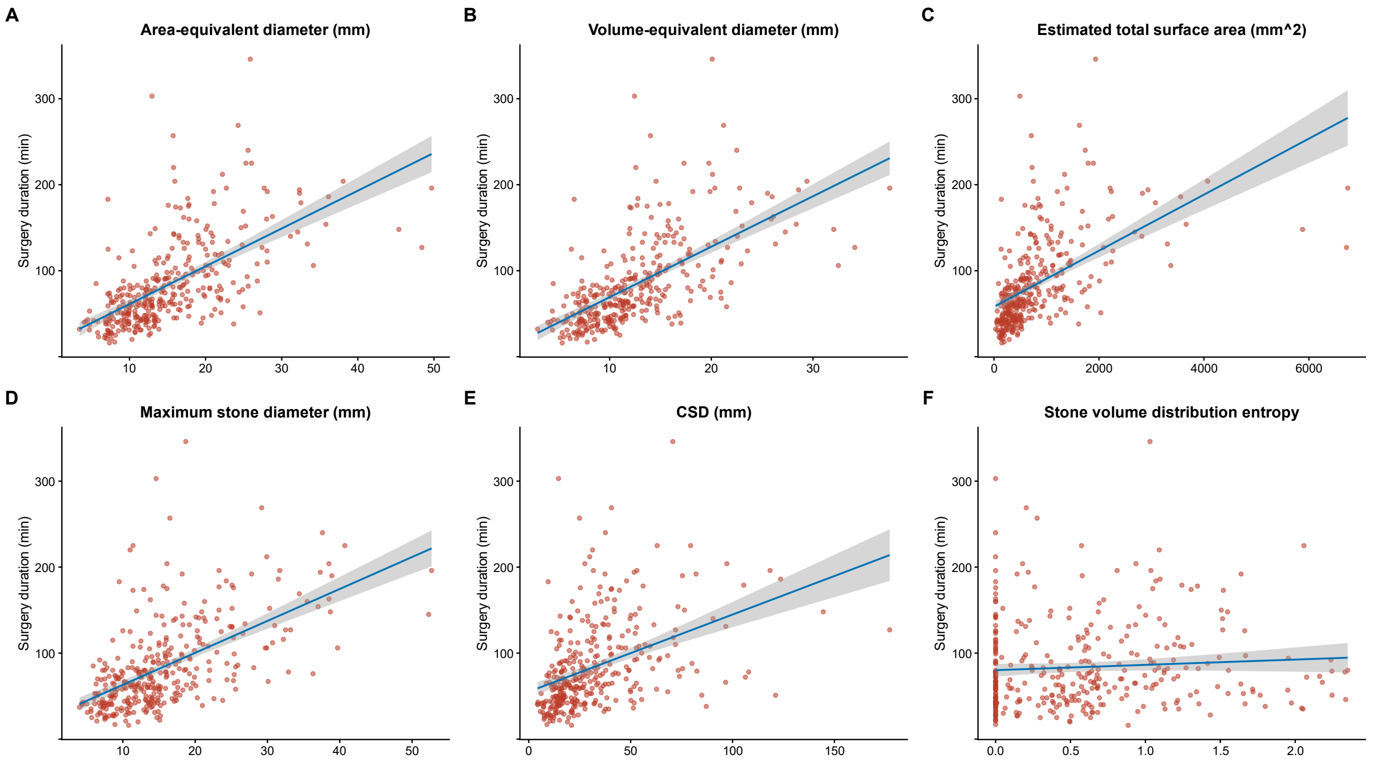

Figure S3. Operative-time prediction via CT-derived stone burden metrics.

(A) Area-equivalent diameter; (B) volume-equivalent diameter; (C) estimated total surface area; (D) maximum stone diameter; (E) cumulative stone diameter; (F) stone volume distribution entropy. All scatter plots were generated in the whole cohort.

Supplementary Tables

Table S1. Definitions and formulas of CT-derived stone burden metrics.

Metric category	Variable	Code name	Formula or definition	Unit
Conventional one-dimensional burden metrics	Long diameter of stone i	L_i	Long diameter of stone i.	mm
Conventional one-dimensional burden metrics	Short diameter of stone i	S_i	Short diameter of stone i.	mm
Conventional one-dimensional burden metrics	Multiple stones	multiple_stones	Binary indicator for stone number > 1.	binary
Conventional one-dimensional burden metrics	Stone number	stone_number	Number of stones included in CT burden calculation.	count
Conventional one-dimensional burden metrics	Maximum stone diameter (mm)	max_stone_diameter_mm	max(Li).	mm
Conventional one-dimensional burden metrics	Cumulative stone diameter (mm)	cumulative_stone_diameter_mm	sum(L_i).	mm
Conventional one-dimensional burden metrics	Length-only area-equivalent diameter (mm)	equivalent_diameter_from_lengths_area_mm	sqrt(sum(L_i^2)), using long diameters only.	mm
Conventional one-dimensional burden metrics	Length-only volume-equivalent diameter (mm)	equivalent_diameter_from_lengths_volume_mm	(sum(L_i^3))^(1/3), using long diameters only.	mm
Conventional one-dimensional burden metrics	Long diameter of stone with largest estimated area (mm)	largest_area_stone_diameter_mm	Long diameter of the stone selected by the largest estimated area.	mm
Conventional one-dimensional burden metrics	Long diameter of stone with largest estimated volume (mm)	largest_volume_stone_diameter_mm	Long diameter of the stone selected by the largest estimated volume.	mm
Two-dimensional burden metrics	Total stone area (mm^2)	total_stone_area_mm2	sum(pi * L_i * S_i / 4).	mm^2
Two-dimensional burden metrics	Area-equivalent diameter (mm)	equivalent_area_diameter_mm	2 * sqrt(total stone area / pi).	mm
Two-dimensional burden metrics	Estimated area of stone with largest long diameter (mm^2)	largest_diameter_stone_area_mm2	Estimated area of the stone selected by the largest long diameter.	mm^2
Two-dimensional burden metrics	Estimated area of stone with largest estimated area (mm^2)	largest_area_stone_area_mm2	Estimated area of the stone selected by the largest estimated area.	mm^2
Two-dimensional burden metrics	Estimated area of stone with largest estimated volume (mm^2)	largest_volume_stone_area_mm2	Estimated area of the stone selected by the largest estimated volume.	mm^2
Estimated three-dimensional burden metrics	Estimated total stone volume (mm^3)	estimated_total_stone_volume_mm3	sum(pi * L_i * S_i^2 / 6).	mm^3
Estimated three-dimensional burden metrics	Volume-equivalent diameter (mm)	equivalent_volume_diameter_mm	(6 * estimated total stone volume / pi)^(1/3).	mm
Estimated three-dimensional burden metrics	Estimated total surface area (mm^2)	estimated_total_surface_area_mm2	sum(SA_i), where SA_i = πS_i^2/2 × [1 + L_i/(S_i e_i) × arcsin(e_i)] and e_i = sqrt(1 − S_i^2/L_i^2); if L_i = S_i, SA_i = πL_i^2.	mm^2
Estimated three-dimensional burden metrics	Estimated volume of stone with largest long diameter (mm^3)	largest_diameter_stone_volume_mm3	Estimated volume of the stone selected by the largest long diameter.	mm^3
Estimated three-	Estimated surface area of stone	largest_diameter_s	Estimated spheroid surface area of the stone selected by the	mm^2

Metric category	Variable	Code name	Formula or definition	Unit
dimensional burden metrics	with largest long diameter (mm^2)	tone_surface_area_mm2	largest long diameter.	
Estimated three-dimensional burden metrics	Estimated volume of stone with largest estimated area (mm^3)	largest_area_stone_volume_mm3	Estimated volume of the stone selected by the largest estimated area.	mm^3
Estimated three-dimensional burden metrics	Estimated surface area of stone with largest estimated area (mm^2)	largest_area_stone_surface_area_mm2	Estimated spheroid surface area of the stone selected by the largest estimated area.	mm^2
Estimated three-dimensional burden metrics	Estimated volume of stone with largest estimated volume (mm^3)	largest_volume_stone_volume_mm3	Estimated volume of the stone selected by the largest estimated volume.	mm^3
Estimated three-dimensional burden metrics	Estimated surface area of stone with largest estimated volume (mm^2)	largest_volume_stone_surface_area_mm2	Estimated spheroid surface area of the stone selected by the largest estimated volume.	mm^2
Morphological and distribution-complexity metrics	Maximum stone aspect ratio	max_stone_aspect_ratio	Maximum Li/Si across stones.	unitless
Morphological and distribution-complexity metrics	Volume-weighted mean aspect ratio	volume_weighted_mean_aspect_ratio	sum(volume_i * aspect_ratio_i) / sum(volume_i).	unitless
Morphological and distribution-complexity metrics	Estimated surface-volume ratio	estimated_surface_volume_ratio	Estimated total surface area / estimated total stone volume.	unitless
Morphological and distribution-complexity metrics	Dominant stone diameter fraction	dominant_stone_diameter_fraction	Largest stone diameter / cumulative stone diameter.	unitless
Morphological and distribution-complexity metrics	Dominant stone area fraction	dominant_stone_area_fraction	Largest stone area / total stone area.	unitless
Morphological and distribution-complexity metrics	Dominant stone volume fraction	dominant_stone_volume_fraction	Largest stone volume / total stone volume.	unitless
Morphological and distribution-complexity metrics	Stone volume distribution entropy	stone_volume_distribution_entropy	-sum(p_i * ln(p_i)), where p_i is stone_i volume / total volume.	unitless
Morphological and distribution-complexity metrics	Normalized stone volume entropy	normalized_stone_volume_entropy	Stone volume entropy / ln(stone number); 0 for single-stone cases.	unitless
Morphological and distribution-complexity metrics	Aspect ratio of stone with largest long diameter	largest_diameter_stone_aspect_ratio	Long-to-short diameter ratio of the stone selected by the largest long diameter.	unitless
Morphological and distribution-complexity metrics	Aspect ratio of stone with largest estimated area	largest_area_stone_aspect_ratio	Long-to-short diameter ratio of the stone selected by the largest estimated area.	unitless
Morphological and distribution-complexity metrics	Aspect ratio of stone with largest estimated volume	largest_volume_stone_aspect_ratio	Long-to-short diameter ratio of the stone selected by the largest estimated volume.	unitless
Spatial distribution metrics	Bilateral stones	bilateral_stones	Binary indicator for bilateral renal/upper-tract stone	binary

Metric category	Variable	Code name	Formula or definition	Unit
			involvement.	
Spatial distribution metrics	Lower calyx involvement	lower_calyx_involved	Binary indicator for lower calyx involvement, including manually confirmed staghorn extension when applicable.	binary
Spatial distribution metrics	Staghorn stone	staghorn_stone	Binary indicator for staghorn stone morphology.	binary
Spatial distribution metrics	Stone site count	stone_site_count	Number of anatomical sites involved by stones.	count
CT attenuation metrics	Maximum stone attenuation (HU)	max_stone_hu	Maximum HU among stones with available CT attenuation.	HU
CT attenuation metrics	Mean stone attenuation (HU)	mean_stone_hu	Arithmetic mean HU among stones with available CT attenuation.	HU
CT attenuation metrics	Diameter-weighted mean stone attenuation (HU)	diameter_weighted_mean_stone_hu	sum(L_i * HU_i) / sum(L_i).	HU
CT attenuation metrics	Area-weighted mean stone attenuation (HU)	area_weighted_mean_stone_hu	sum(area_i * HU_i) / sum(area_i).	HU
CT attenuation metrics	Volume-weighted mean stone attenuation (HU)	volume_weighted_mean_stone_hu	sum(volume_i * HU_i) / sum(volume_i).	HU
CT attenuation metrics	Stone attenuation of stone with largest long diameter (HU)	largest_diameter_stone_hu	CT attenuation of the stone selected by the largest long diameter.	HU
CT attenuation metrics	Stone attenuation of stone with largest estimated area (HU)	largest_area_stone_hu	CT attenuation of the stone selected by the largest estimated area.	HU
CT attenuation metrics	Stone attenuation of stone with largest estimated volume (HU)	largest_volume_stone_hu	CT attenuation of the stone selected by the largest estimated volume.	HU

Table S2. Baseline characteristics of the training and validation sets.

Variable	Overall	Training (n=247)	Validation (n=105)	Statistic (t/Z/χ2)	P
Operative time (min)	70.00[47.00,106.00]	73.00[48.50,106.00]	64.00[42.00,106.00]	1.469	0.142
Age (years)	54.37(±12.37)	54.46(±12.39)	54.16(±12.36)	0.205	0.838
Sex				1.156	0.282
Female	122(34.66)	90(36.44)	32(30.48)		
Male	230(65.34)	157(63.56)	73(69.52)		
BMI (kg/m²)	25.25[22.90,27.34]	25.25[22.95,26.70]	25.22[22.86,28.04]	0.994	0.320
eGFR (mL/min/1.73 m²)	100.30[86.20,107.73]	100.60[86.75,108.45]	98.70[84.10,105.70]	0.830	0.407
CKD G stage				3.817	0.576
G1	249(70.74)	177(71.66)	72(68.57)		
G2	78(22.16)	51(20.65)	27(25.71)		
G3a	11(3.12)	7(2.83)	4(3.81)		
G3b	8(2.27)	6(2.43)	2(1.90)		
G4	5(1.42)	5(2.02)	0(0.00)		
G5	1(0.28)	1(0.40)	0(0.00)		
Hyperuricemia or gout				0.223	0.636
No	252(71.59)	175(70.85)	77(73.33)		
Yes	100(28.41)	72(29.15)	28(26.67)		
Renal tubular acidosis				<0.001	0.993
No	347(98.58)	244(98.79)	103(98.10)		
Yes	5(1.42)	3(1.21)	2(1.90)		
Sjogren syndrome				<0.001	1.000
No	346(98.30)	243(98.38)	103(98.10)		
Yes	6(1.70)	4(1.62)	2(1.90)		
Complex urological anatomy				0.326	0.568
No	304(86.36)	215(87.04)	89(84.76)		
Yes	48(13.64)	32(12.96)	16(15.24)		
Scope outer diameter (Fr)	7.50[7.50,7.50]	7.50[7.50,7.50]	7.50[7.50,8.60]	1.078	0.151
Surgical position				2.826	0.587
GMSV	4(1.14)	3(1.21)	1(0.95)		
Left lateral decubitus	1(0.28)	1(0.40)	0(0.00)		
Lithotomy	100(28.41)	70(28.34)	30(28.57)		
Supine	1(0.28)	0(0.00)	1(0.95)		
Supine split leg	246(69.89)	173(70.04)	73(69.52)		
Stone site group				2.233	0.135
Kidney	282(80.11)	203(82.19)	79(75.24)		
Kidney and ureter	70(19.89)	44(17.81)	26(24.76)		
Stone laterality				0.974	0.615
Bilateral	38(10.80)	27(10.93)	11(10.48)		
Left	190(53.98)	137(55.47)	53(50.48)		
Right	124(35.23)	83(33.60)	41(39.05)		
Stone number	2.00[1.00,4.00]	2.00[1.00,4.00]	2.00[1.00,4.00]	0.355	0.718
Maximum stone diameter (mm)	13.90[10.15,18.50]	14.10[10.75,18.40]	13.00[9.20,18.50]	1.725	0.085
Cumulative stone diameter (mm)	24.95[15.67,39.62]	25.50[16.00,41.95]	23.90[15.00,37.50]	1.302	0.193
Equivalent diameter from lengths area mm	16.86[12.38,23.47]	17.25[13.21,24.61]	16.44[11.74,22.00]	1.676	0.094

Variable	Overall	Training (n=247)	Validation (n=105)	Statistic (t/Z/χ2)	P
Equivalent diameter from lengths volume mm	15.11[11.09,20.44]	15.40[11.83,20.68]	14.74[10.16,20.06]	1.705	0.088
Total stone area (mm²)	143.27[78.35,279.68]	150.75[81.79,300.21]	132.20[63.99,249.26]	1.645	0.100
Area-equivalent diameter (mm)	13.51[9.99,18.87]	13.85[10.20,19.55]	12.97[9.03,17.81]	1.645	0.100
Estimated total stone volume (mm³)	786.40[326.14,1740.40]	813.95[357.67,1750.11]	644.71[239.91,1619.33]	1.629	0.104
Volume-equivalent diameter (mm)	11.45[8.54,14.92]	11.58[8.81,14.95]	10.72[7.71,14.57]	1.629	0.104
Max stone aspect ratio	1.81[1.53,2.10]	1.82[1.54,2.10]	1.79[1.52,2.10]	0.558	0.578
Volume weighted mean aspect ratio	1.49[1.30,1.77]	1.49[1.31,1.80]	1.49[1.26,1.70]	1.132	0.258
Estimated total surface area (mm²)	524.97[287.19,1003.80]	550.68[291.12,1069.34]	465.84[226.59,904.08]	1.611	0.107
Estimated surface-volume ratio	0.69[0.51,0.89]	0.67[0.50,0.87]	0.75[0.54,0.98]	1.556	0.120
Dominant stone diameter fraction	0.58[0.40,1.00]	0.57[0.39,1.00]	0.60[0.41,1.00]	0.537	0.587
Dominant stone area fraction	0.74[0.50,1.00]	0.74[0.50,1.00]	0.75[0.52,1.00]	0.451	0.649
Dominant stone volume fraction	0.85[0.60,1.00]	0.84[0.59,1.00]	0.86[0.62,1.00]	0.424	0.669
Stone volume distribution entropy	0.48[0.00,0.93]	0.48[0.00,0.94]	0.48[0.00,0.87]	0.465	0.639
Normalized stone volume entropy	0.46[0.00,0.74]	0.47[0.00,0.75]	0.44[0.00,0.73]	0.411	0.678
Stone site count	2.00[1.00,3.00]	2.00[1.00,3.00]	2.00[1.00,3.00]	0.357	0.707
Maximum stone attenuation (HU)	1065.00[1003.00,1208.50]	1069.00[1004.00,1204.00]	1059.00[1003.00,1225.00]	0.373	0.710
Mean stone attenuation (HU)	1004.50[914.75,1063.00]	1003.00[915.00,1063.00]	1009.67[873.67,1063.00]	0.096	0.924
Diameter-weighted mean stone attenuation (HU)	1013.46[935.20,1075.39]	1010.00[934.51,1078.47]	1017.90[935.78,1063.00]	0.581	0.562
Area-weighted mean stone attenuation (HU)	1022.00[938.72,1099.46]	1018.94[942.43,1114.32]	1026.00[937.72,1091.53]	0.491	0.624
Volume-weighted mean stone attenuation (HU)	1026.52[946.11,1104.22]	1022.74[948.74,1114.57]	1030.00[944.00,1100.43]	0.42	0.675
Estimated area of stone with largest long diameter (mm²)	95.70[51.01,167.48]	99.89[56.06,169.03]	82.70[40.29,154.90]	1.824	0.068
Estimated volume of stone with largest long diameter (mm³)	546.82[223.97,1292.95]	593.38[242.91,1368.63]	477.13[175.35,1062.90]	1.894	0.058
Estimated surface area of stone with largest long diameter (mm²)	343.79[185.80,593.38]	365.49[202.32,617.88]	299.02[152.28,541.73]	1.858	0.063
Aspect ratio of stone with largest long diameter	1.54[1.29,1.90]	1.54[1.29,1.94]	1.52[1.28,1.85]	0.424	0.672
Stone attenuation of stone with largest long diameter (HU)	1038.00[943.75,1147.00]	1038.00[947.00,1152.00]	1038.00[936.00,1122.00]	0.891	0.373
Long diameter of stone with largest estimated area (mm)	13.85[9.97,18.35]	14.10[10.45,18.25]	12.90[9.00,18.50]	1.813	0.070
Estimated area of stone with largest estimated area (mm²)	95.70[52.86,167.48]	99.89[59.14,169.03]	82.70[42.73,155.02]	1.719	0.086
Estimated volume of stone with largest estimated area (mm³)	550.51[238.81,1296.55]	599.83[266.67,1368.63]	481.29[175.62,1073.01]	1.75	0.080
Estimated surface area of stone with largest estimated area (mm²)	344.08[192.10,597.11]	365.49[210.78,617.88]	301.60[155.33,553.97]	1.721	0.085
Aspect ratio of stone with largest estimated area	1.48[1.24,1.82]	1.51[1.26,1.87]	1.43[1.21,1.81]	1.107	0.269
Stone attenuation of stone with largest	1039.00[944.00,1147.00]	1039.00[947.00,1148.00]	1041.00[944.00,1135.00]	0.663	0.508

Variable	Overall	Training (n=247)	Validation (n=105)	Statistic ($t/Z/\chi2$)	P
estimated area (HU)					
Long diameter of stone with largest estimated volume (mm)	13.80[9.80,18.22]	14.10[10.40,18.25]	12.90[9.00,18.00]	1.832	0.067
Estimated area of stone with largest estimated volume (mm^2)	95.70[52.59,165.86]	99.89[59.14,169.03]	82.70[42.73,155.02]	1.732	0.083
Estimated volume of stone with largest estimated volume (mm^3)	550.51[240.57,1296.55]	599.83[271.08,1368.63]	481.29[175.62,1083.26]	1.752	0.08
Estimated surface area of stone with largest estimated volume (mm^2)	344.08[192.10,593.38]	365.49[210.78,617.88]	301.60[155.33,553.97]	1.726	0.084
Aspect ratio of stone with largest estimated volume	1.48[1.24,1.81]	1.50[1.26,1.81]	1.42[1.21,1.76]	1.194	0.233
Stone attenuation of stone with largest estimated volume (HU)	1039.50[947.75,1150.00]	1039.00[951.50,1152.00]	1041.00[944.00,1135.00]	0.734	0.463

Table S3. Comparison of included and excluded RIRS episodes.

Characteristic	Included (n=352)	Excluded (n=133)	P value
Age, years	55.0 [45.8, 63.0]	55.0 [46.0, 65.0]	0.761
Sex			0.522
female	122 (34.7)	42 (31.6)	
male	230 (65.3)	91 (68.4)	
BMI, kg/m²	25.2 [22.9, 27.3]	24.9 [22.6, 27.2]	0.643
eGFR, mL/min/1.73 m²	100.3 [86.2, 107.7]	98.0 [79.4, 107.9]	0.349
Hypertension	156 (44.3)	63 (47.4)	0.547
Diabetes mellitus	66 (18.8)	36 (27.1)	0.045
Coronary heart disease	35 (9.9)	17 (12.8)	0.367
Preoperative D-J stent	101 (28.7)	58 (43.6)	0.002
Positive preoperative urine culture	148 (45.0)	59 (47.2)	0.894
Preoperative pyuria	249 (70.7)	95 (72.0)	0.355
Positive preoperative urine nitrite	27 (7.7)	6 (4.5)	0.134
Stone site group			<0.001
Kidney	282 (80.1)	64 (48.1)	
Kidney and ureter	70 (19.9)	7 (5.3)	
Multi or other	0 (0.0)	1 (0.8)	
Ureter	0 (0.0)	61 (45.9)	
Stone laterality			0.746
Bilateral	38 (10.8)	12 (9.0)	
Left	190 (54.0)	70 (52.6)	
Right	124 (35.2)	51 (38.3)	
Lower calyx involvement	275 (78.1)	51 (38.3)	<0.001
Staghorn stone	56 (15.9)	13 (9.8)	0.084
Multiple stones	252 (71.6)	49 (36.8)	<0.001
Surgical position			0.909
Gmsv	4 (1.1)	2 (1.5)	
Left lateral decubitus	1 (0.3)	0 (0.0)	
Lithotomy	100 (28.4)	36 (27.1)	
Supine	1 (0.3)	0 (0.0)	
Supine split leg	246 (69.9)	95 (71.4)	
Ureteroscope outer diameter, Fr	7.5 [7.5, 7.5]	7.5 [7.5, 7.5]	0.343
Operative time, min	70.0 [47.0, 106.0]	65.0 [40.0, 94.0]	0.090
Stone-free status	211 (59.9)	103 (77.4)	<0.001

Table S4. Interobserver reproducibility of CT-derived stone measurements and burden metrics.

Measurement or metric	n	ICC (95% CI)	P value	Pearson r	Spearman rho	Original mean	Repeated mean	Mean difference
Stone number	30	0.997 (0.994-0.999)	<0.001	0.998	1.000	2.60	2.57	-0.033
Maximum stone diameter, mm	30	0.989 (0.978-0.995)	<0.001	0.989	0.964	16.52	16.52	-0.003
Short diameter of the stone with largest long diameter, mm	30	0.969 (0.937-0.985)	<0.001	0.972	0.924	10.39	10.30	-0.090
CSD, mm	30	0.998 (0.995-0.999)	<0.001	0.998	0.986	26.60	26.70	0.107
Total stone area, mm^2	30	0.993 (0.986-0.997)	<0.001	0.994	0.966	195.5	198.4	2.88
Area-equivalent diameter, mm	30	0.992 (0.983-0.996)	<0.001	0.992	0.966	14.58	14.64	0.057
Estimated total stone volume, mm^3	30	0.987 (0.973-0.994)	<0.001	0.991	0.972	1569.5	1624.5	55.00
Volume-equivalent diameter, mm	30	0.988 (0.976-0.994)	<0.001	0.988	0.972	12.60	12.64	0.047
Estimated total stone surface area, mm^2	30	0.993 (0.985-0.996)	<0.001	0.994	0.960	704.2	715.9	11.66
Stone volume distribution entropy	30	0.997 (0.994-0.999)	<0.001	0.997	0.999	0.302	0.305	0.003
Maximum stone attenuation, HU	30	0.977 (0.947-0.990)	<0.001	0.984	0.934	1084.2	1059.4	-24.83
Mean stone attenuation, HU	30	0.986 (0.968-0.994)	<0.001	0.988	0.877	937.2	919.5	-17.71

Table S5. Complete univariable logistic regression analysis for stone-free status.

Variable	Comparison / unit	OR (95% CI)	P	AUC (Training)	Brier (Training)	AUC (Validation)	Brier (Validation)
Area-equivalent diameter (mm)	per 10 mm	0.170(0.099-0.293)	<0.001	0.773	0.185	0.755	0.199
Length-only area-equivalent diameter (mm)	per 10 mm	0.294(0.200-0.433)	<0.001	0.761	0.190	0.745	0.201
Volume-equivalent diameter (mm)	per 10 mm	0.116(0.058-0.230)	<0.001	0.752	0.190	0.725	0.205
Length-only volume-equivalent diameter (mm)	per 10 mm	0.268(0.174-0.414)	<0.001	0.736	0.195	0.709	0.208
Estimated total surface area (mm²)	per 100 mm²	0.836(0.787-0.887)	<0.001	0.771	0.186	0.757	0.202
Total stone area (mm²)	per 100 mm²	0.528(0.426-0.653)	<0.001	0.773	0.187	0.755	0.202
Cumulative stone diameter (mm)	per 10 mm	0.655(0.563-0.763)	<0.001	0.757	0.201	0.755	0.202
Long diameter of stone with largest estimated volume (mm)	per 10 mm	0.311(0.203-0.476)	<0.001	0.693	0.204	0.647	0.220
Maximum stone diameter (mm)	per 10 mm	0.310(0.202-0.475)	<0.001	0.693	0.204	0.653	0.219
Estimated surface area of stone with largest estimated volume (mm²)	per 100 mm²	0.779(0.711-0.853)	<0.001	0.704	0.196	0.660	0.215
Estimated surface area of stone with largest estimated area (mm²)	per 100 mm²	0.779(0.711-0.853)	<0.001	0.704	0.196	0.659	0.215
Long diameter of stone with largest estimated area (mm)	per 10 mm	0.313(0.205-0.479)	<0.001	0.690	0.204	0.652	0.219
Estimated area of stone with largest estimated volume (mm²)	per 100 mm²	0.416(0.301-0.574)	<0.001	0.703	0.196	0.660	0.215
Estimated surface area of stone with largest long diameter (mm²)	per 100 mm²	0.779(0.711-0.854)	<0.001	0.704	0.197	0.661	0.216
Estimated area of stone with largest estimated area (mm²)	per 100 mm²	0.416(0.301-0.574)	<0.001	0.703	0.196	0.660	0.215
Estimated area of stone with largest long diameter (mm²)	per 100 mm²	0.416(0.301-0.576)	<0.001	0.703	0.197	0.663	0.216
Estimated total stone volume (mm³)	per 1000 mm³	0.523(0.409-0.668)	<0.001	0.752	0.191	0.725	0.208
Staghorn stone	Yes vs No	0.149(0.067-0.331)	<0.001	0.621	0.215	0.583	0.231
Estimated volume of stone with largest estimated area (mm³)	per 1000 mm³	0.455(0.331-0.624)	<0.001	0.705	0.195	0.660	0.215
Estimated volume of stone with largest estimated	per 1000 mm³	0.455(0.331-0.624)	<0.001	0.704	0.195	0.661	0.215

Variable	Comparison / unit	OR (95% CI)	P	AUC (Training)	Brier (Training)	AUC (Validation)	Brier (Validation)
volume (mm³)							
Estimated volume of stone with largest long diameter (mm³)	per 1000 mm³	0.454(0.329-0.626)	<0.001	0.704	0.196	0.662	0.217
Stone site count	per unit	0.564(0.416-0.765)	<0.001	0.628	0.226	0.588	0.235
Estimated surface-volume ratio	per unit	5.677(2.213-14.566)	<0.001	0.668	0.224	0.638	0.231
Stone volume distribution entropy	per unit	0.454(0.287-0.719)	<0.001	0.618	0.228	0.639	0.220
Lower calyx involvement	Yes vs No	0.326(0.158-0.672)	0.001	0.583	0.231	0.540	0.241
Dominant stone diameter fraction	per unit	4.567(1.784-11.689)	0.002	0.622	0.230	0.638	0.226
Stone number	per unit	0.863(0.786-0.947)	0.002	0.631	0.229	0.625	0.218
Positive preoperative urine nitrite	Yes vs No	0.264(0.109-0.637)	0.002	0.568	0.231	0.544	0.239
Dominant stone area fraction	per unit	4.891(1.751-13.667)	0.002	0.611	0.231	0.641	0.222
Dominant stone volume fraction	per unit	5.097(1.668-15.579)	0.004	0.606	0.232	0.646	0.220
Sjogren syndrome	Yes vs No	0.000(0.000-Inf)	0.006	0.520	0.234	0.524	0.233
Renal tubular acidosis	Yes vs No	0.000(0.000-Inf)	0.019	0.515	0.236	0.524	0.233
Multiple stones	Yes vs No	0.511(0.281-0.930)	0.025	0.565	0.235	0.548	0.238
Maximum stone attenuation (HU)	per 100 HU	0.909(0.829-0.997)	0.044	0.572	0.236	0.538	0.240
Normalized stone volume entropy	per unit	0.467(0.221-0.985)	0.046	0.574	0.236	0.616	0.232
Surgical position	supine_split_leg vs gmsv	9109375.770(0.000-Inf)	0.059	0.525	0.234	0.593	0.246
Surgical position	lithotomy vs gmsv	8636719.480(0.000-Inf)	0.059	0.525	0.234	0.593	0.246
Surgical position	left_lateral_decubitus vs gmsv	1.000(0.000-Inf)	0.059	0.525	0.234	0.593	0.246
Maximum stone aspect ratio	per unit	0.704(0.469-1.055)	0.089	0.583	0.237	0.597	0.240
Mean stone attenuation (HU)	per 100 HU	0.907(0.810-1.017)	0.095	0.557	0.237	0.491	0.245
Sex	Male vs Female	0.638(0.372-1.094)	0.100	0.551	0.238	0.496	0.243
Complex urological anatomy	Yes vs No	0.544(0.258-1.148)	0.110	0.535	0.238	0.492	0.244
Stone site group	kidney_and_ureter vs kidney	1.753(0.866-3.547)	0.110	0.539	0.238	0.508	0.242
Diameter-weighted mean stone attenuation (HU)	per 100 HU	0.911(0.806-1.030)	0.135	0.553	0.238	0.478	0.245
Stone attenuation of stone with largest long diameter (HU)	per 100 HU	0.938(0.852-1.034)	0.197	0.553	0.239	0.567	0.240
Area-weighted mean stone attenuation (HU)	per 100 HU	0.926(0.823-1.041)	0.199	0.545	0.238	0.478	0.244
Coronary heart disease	Yes vs No	0.584(0.255-1.338)	0.204	0.525	0.239	0.520	0.239

Variable	Comparison / unit	OR (95% CI)	P	AUC (Training)	Brier (Training)	AUC (Validation)	Brier (Validation)
BMI (kg/m²)	per unit	0.958(0.895-1.027)	0.226	0.512	0.239	0.514	0.240
Volume-weighted mean stone attenuation (HU)	per 100 HU	0.935(0.836-1.046)	0.240	0.541	0.239	0.486	0.243
Stone attenuation of stone with largest estimated area (HU)	per 100 HU	0.945(0.858-1.040)	0.246	0.547	0.239	0.515	0.242
Stone laterality	right vs left	0.628(0.361-1.094)	0.259	0.555	0.238	0.457	0.248
Stone laterality	bilateral vs left	0.810(0.348-1.883)	0.259	0.555	0.238	0.457	0.248
Scope outer diameter (Fr)	8.6 vs 7.5	0.629(0.344-1.153)	0.344	0.545	0.237	0.631	0.229
Scope outer diameter (Fr)	7.5/8.6 vs 7.5	1.214(0.108-13.629)	0.344	0.545	0.237	0.631	0.229
Scope outer diameter (Fr)	6.3 vs 7.5	1285391.268(0.000-Inf)	0.344	0.545	0.237	0.631	0.229
Stone attenuation of stone with largest estimated volume (HU)	per 100 HU	0.955(0.867-1.053)	0.354	0.539	0.239	0.518	0.241
History of malignancy	Yes vs No	1.482(0.582-3.778)	0.401	0.515	0.239	0.476	0.243
eGFR (mL/min/1.73 m²)	per unit	1.004(0.993-1.016)	0.451	0.535	0.240	0.474	0.240
Aspect ratio of stone with largest estimated volume	per unit	0.838(0.518-1.355)	0.471	0.519	0.240	0.541	0.240
Age (years)	per unit	1.007(0.987-1.028)	0.493	0.527	0.240	0.587	0.238
Hyperuricemia or gout	Yes vs No	0.841(0.482-1.467)	0.541	0.518	0.240	0.496	0.241
CKD G stage by eGFR	G2 vs G1	0.633(0.338-1.186)	0.593	0.551	0.237	0.522	0.241
CKD G stage by eGFR	G3b vs G1	0.609(0.119-3.105)	0.593	0.551	0.237	0.522	0.241
CKD G stage by eGFR	G3a vs G1	1.523(0.287-8.071)	0.593	0.551	0.237	0.522	0.241
CKD G stage by eGFR	G4 vs G1	0.914(0.149-5.610)	0.593	0.551	0.237	0.522	0.241
CKD G stage by eGFR	G5 vs G1	1290164.168(0.000-Inf)	0.593	0.551	0.237	0.522	0.241
Hypertension	Yes vs No	0.878(0.526-1.464)	0.618	0.516	0.240	0.552	0.239
Preoperative pyuria	Yes vs No	0.845(0.431-1.656)	0.622	0.513	0.242	0.607	0.241
Diabetes mellitus	Yes vs No	0.856(0.442-1.655)	0.644	0.512	0.240	0.504	0.240
Aspect ratio of stone with largest estimated area	per unit	0.899(0.560-1.442)	0.658	0.512	0.240	0.553	0.239
Ongoing antithrombotic therapy	Yes vs No	0.664(0.092-4.796)	0.686	0.503	0.240	0.492	0.241
Aspect ratio of stone with largest long diameter	per unit	0.926(0.584-1.467)	0.743	0.513	0.240	0.540	0.240
CKD G3 or higher	Yes vs No	1.160(0.440-3.056)	0.763	0.505	0.240	0.488	0.240
Volume-weighted mean aspect ratio	per unit	0.922(0.533-1.595)	0.772	0.521	0.240	0.522	0.240
Preoperative D-J stent	Yes vs No	1.057(0.598-1.866)	0.849	0.506	0.240	0.377	0.242
Positive preoperative urine culture	Yes vs No	0.978(0.576-1.661)	0.934	0.503	0.241	0.456	0.244
Bilateral stones	Yes vs No	0.970(0.430-2.188)	0.941	0.502	0.240	0.552	0.240

Table S6. Spearman's correlation clustering and retained candidate variables for stone-free status prediction.

Cluster	Rank in cluster	Retained: top 2 and P<0.05	Variable	Unit
1	1	No	Area-weighted mean stone attenuation (HU)	HU
1	2	No	Volume-weighted mean stone attenuation (HU)	HU
2	1	Yes	Cumulative stone diameter (mm)	mm
3	1	No	Mean stone attenuation (HU)	HU
3	2	No	Diameter-weighted mean stone attenuation (HU)	HU
4	1	Yes	Stone volume distribution entropy	unitless
4	2	Yes	Dominant stone area fraction	unitless
4	3	No	Dominant stone volume fraction	unitless
4	4	No	Normalized stone volume entropy	unitless
5	1	Yes	Dominant stone diameter fraction	unitless
5	2	Yes	Stone number	count
6	1	Yes	Area-equivalent diameter (mm)	mm
6	2	Yes	Length-only area-equivalent diameter (mm)	mm
6	3	No	Volume-equivalent diameter (mm)	mm
6	4	No	Length-only volume-equivalent diameter (mm)	mm
6	5	No	Estimated total surface area (mm^2)	mm^2
6	6	No	Total stone area (mm^2)	mm^2
6	7	No	Estimated total stone volume (mm^3)	mm^3
7	1	Yes	Long diameter of stone with largest estimated volume (mm)	mm
7	2	Yes	Maximum stone diameter (mm)	mm
7	3	No	Estimated surface area of stone with largest estimated volume (mm^2)	mm^2
7	4	No	Estimated surface area of stone with largest estimated area (mm^2)	mm^2
7	5	No	Long diameter of stone with largest estimated area (mm)	mm
7	6	No	Estimated area of stone with largest estimated volume (mm^2)	mm^2
7	7	No	Estimated surface area of stone with largest long diameter (mm^2)	mm^2
7	8	No	Estimated area of stone with largest estimated area (mm^2)	mm^2
7	9	No	Estimated area of stone with largest long diameter (mm^2)	mm^2
7	10	No	Estimated volume of stone with largest estimated area (mm^3)	mm^3
7	11	No	Estimated volume of stone with largest estimated volume (mm^3)	mm^3
7	12	No	Estimated volume of stone with largest long diameter (mm^3)	mm^3
7	13	No	Estimated surface-volume ratio	unitless
8	1	No	Aspect ratio of stone with largest estimated volume	unitless
8	2	No	Aspect ratio of stone with largest estimated area	unitless
8	3	No	Aspect ratio of stone with largest long diameter	unitless
8	4	No	Volume-weighted mean aspect ratio	unitless
9	1	No	Stone attenuation of stone with largest long diameter (HU)	HU
9	2	No	Stone attenuation of stone with largest estimated area (HU)	HU
9	3	No	Stone attenuation of stone with largest estimated volume (HU)	HU
10	1	No	Maximum stone aspect ratio	unitless
11	1	Yes	Maximum stone attenuation (HU)	HU
12	1	Yes	Stone site count	count
13	1	No	Bilateral stones	binary
14	1	Yes	Lower calyx involvement	binary

15	1	Yes	Multiple stones	binary
16	1	Yes	Staghorn stone	binary

Table S7. Full model equations, AUC values, and Brier scores with 95% CIs, calibration intercepts, and slopes.

Item	Main model	CSD model
Equation	logit(P stone-free) = 3.026 - 0.140 x equivalent_area_diameter_mm - 0.977 x staghorn_stone - 0.486 x stone_volume_distribution_entropy	logit(P stone-free) = 1.840 - 0.037 x cumulative_stone_diameter_mm - 1.465 x staghorn_stone
Training AUC (95% CI)	0.794 (0.737-0.851)	0.782 (0.724-0.840)
Validation AUC (95% CI)	0.779 (0.690-0.867)	0.761 (0.670-0.852)
Training Brier (95% CI)	0.180 (0.155-0.206)	0.189 (0.165-0.214)
Validation Brier (95% CI)	0.194 (0.155-0.234)	0.203 (0.164-0.242)
Training calibration intercept	-0.000	-0.000
Training calibration slope	1.000	1.000
Validation calibration intercept	-0.128	-0.060
Validation calibration slope	0.932	0.903

Table S8. Repeated patient-level full-pipeline validation for stone-free status prediction.

Item	Result
Validation setting	500 repeated patient-level 7:3 training-validation splits
Patient-level split	Yes; all episodes from the same patient were assigned to the same split
Pipeline repeated within each training resample	Yes; univariable screening, Spearman |rho|≥0.85 clustering, cluster-wise retention, stepwise logistic regression, and validation-set performance estimation were repeated
Successful splits	500/500
Full-pipeline validation AUC, median (IQR)	0.761 (0.738-0.787)
Full-pipeline validation Brier score, median (IQR)	0.195 (0.184-0.205)
Fixed main model validation AUC, median (IQR)	0.782 (0.758-0.805)
Fixed main model validation Brier score, median (IQR)	0.187 (0.177-0.197)
Fixed CSD model validation AUC, median (IQR)	0.777 (0.749-0.800)
Fixed CSD model validation Brier score, median (IQR)	0.195 (0.185-0.204)
Retained candidate-pool size, median	8.0
Final variable count, median	3.0
Selection frequency: Area-equivalent diameter (mm)	381/500 (76.2%)
Selection frequency: Stone volume distribution entropy	302/500 (60.4%)
Selection frequency: Staghorn stone	222/500 (44.4%)
Selection frequency: Volume-equivalent diameter (mm)	143/500 (28.6%)

Table S9. Penalized-regression sensitivity analysis and bootstrap selection frequencies.

Item	lambda.min	lambda.1se
Penalty parameter	0.029425	0.207590
No. selected variables	5	1
Selected variables	Area-equivalent diameter; Long diameter of stone with largest estimated volume; Maximum stone attenuation; Stone site count; Staghorn stone	Area-equivalent diameter
Validation AUC	0.760	0.755
Validation Brier score	0.198	0.233
Bootstrap selection frequency, n (%)		
Area-equivalent diameter	489 (97.8%)	378 (75.6%)
Length-only area-equivalent diameter	50 (10.0%)	53 (10.6%)
CSD	4 (0.8%)	6 (1.2%)
Staghorn stone	437 (87.4%)	3 (0.6%)
Long diameter of stone with largest estimated volume	277 (55.4%)	3 (0.6%)
Maximum stone diameter	47 (9.4%)	3 (0.6%)
Maximum stone attenuation	387 (77.4%)	0 (0.0%)
Stone site count	308 (61.6%)	0 (0.0%)
Lower calyx involvement	206 (41.2%)	0 (0.0%)
Stone volume distribution entropy	187 (37.4%)	0 (0.0%)
Stone number	111 (22.2%)	0 (0.0%)
Multiple stones	65 (13.0%)	0 (0.0%)
Dominant stone diameter fraction	38 (7.6%)	0 (0.0%)
Dominant stone area fraction	6 (1.2%)	0 (0.0%)

Table S10. Complete univariable linear regression analysis for operative time in the whole cohort.

Variable	Beta (95% CI)	P	Spearman rho	Spearman P	Training R^2	Validation R^2	Training RMSE	Validation RMSE	Validation MAE
Volume-equivalent diameter (mm)	5.682(4.765-6.600)	<0.001	0.678	<0.001	0.376	0.439	40.929	36.452	26.872
Area-equivalent diameter (mm)	4.284(3.565-5.003)	<0.001	0.669	<0.001	0.358	0.404	41.508	37.559	27.597
Length-only volume-equivalent diameter (mm)	3.614(3.003-4.225)	<0.001	0.638	<0.001	0.354	0.419	41.626	37.086	27.291
Length-only area-equivalent diameter (mm)	3.102(2.567-3.636)	<0.001	0.641	<0.001	0.346	0.400	41.896	37.702	27.913
Maximum stone diameter (mm)	3.599(2.940-4.258)	<0.001	0.591	<0.001	0.319	0.373	42.745	38.540	28.241
Long diameter of stone with largest estimated volume (mm)	3.553(2.895-4.212)	<0.001	0.583	<0.001	0.313	0.361	42.915	38.878	28.467
Long diameter of stone with largest estimated area (mm)	3.560(2.900-4.221)	<0.001	0.582	<0.001	0.313	0.368	42.928	38.680	28.320
Estimated area of stone with largest estimated area (mm²)	0.190(0.153-0.226)	<0.001	0.626	<0.001	0.295	0.350	43.477	39.223	29.312
Estimated area of stone with largest estimated volume (mm²)	0.190(0.153-0.226)	<0.001	0.626	<0.001	0.295	0.349	43.481	39.251	29.324
Estimated surface area of stone with largest estimated area (mm²)	0.054(0.044-0.065)	<0.001	0.627	<0.001	0.294	0.348	43.516	39.272	29.440
Estimated surface area of stone with largest estimated volume (mm²)	0.054(0.044-0.065)	<0.001	0.626	<0.001	0.294	0.349	43.520	39.258	29.430
Estimated area of stone with largest long diameter (mm²)	0.188(0.152-0.225)	<0.001	0.623	<0.001	0.291	0.347	43.613	39.307	29.449
Estimated surface area of stone with largest long diameter (mm²)	0.054(0.043-0.064)	<0.001	0.621	<0.001	0.288	0.344	43.715	39.414	29.646
Total stone area (mm²)	0.112(0.090-0.135)	<0.001	0.669	<0.001	0.286	0.303	43.766	40.613	30.906
Estimated total surface area (mm²)	0.031(0.025-0.038)	<0.001	0.669	<0.001	0.283	0.298	43.861	40.756	31.103
Estimated total stone volume (mm³)	0.008(0.006-0.010)	<0.001	0.678	<0.001	0.230	0.247	45.451	42.227	32.946
Estimated surface-volume ratio	-73.963(-91.259--56.667)	<0.001	-0.586	<0.001	0.223	0.290	45.659	40.996	31.975
Estimated volume of stone with largest estimated area (mm³)	0.011(0.008-0.013)	<0.001	0.624	<0.001	0.222	0.257	45.677	41.934	32.331
Estimated volume of stone with largest estimated volume (mm³)	0.011(0.008-0.013)	<0.001	0.624	<0.001	0.222	0.258	45.679	41.915	32.321
Estimated volume of stone with largest long diameter (mm³)	0.011(0.008-0.013)	<0.001	0.615	<0.001	0.216	0.253	45.873	42.058	32.503
Cumulative stone diameter (mm)	0.893(0.649-1.137)	<0.001	0.530	<0.001	0.174	0.167	47.075	44.411	34.204
Staghorn stone	57.633(41.377-73.889)	<0.001			0.165	0.143	47.338	45.052	34.994
Stone site count	9.139(1.857-16.421)	0.015	0.161	0.011	0.024	-0.053	51.166	49.917	40.559
Stone attenuation of stone with largest estimated volume (HU)	-0.026(-0.050--0.001)	0.041	-0.070	0.275	0.017	0.031	51.353	47.897	39.749
Stone attenuation of stone with largest estimated area (HU)	-0.022(-0.047-0.002)	0.072	-0.052	0.415	0.013	0.022	51.452	48.123	39.911
Dominant stone volume fraction	-24.715(-52.658-3.228)	0.084	-0.142	0.025	0.012	-0.018	51.479	49.082	40.629
Stone attenuation of stone with largest long diameter (HU)	-0.021(-0.045-0.004)	0.100	-0.031	0.622	0.011	0.011	51.508	48.396	40.162

Variable	Beta (95% CI)	P	Spearman rho	Spearman P	Training R^2	Validation R^2	Training RMSE	Validation RMSE	Validation MAE
Dominant stone area fraction	-20.620(-46.052-4.813)	0.113	-0.140	0.028	0.010	-0.018	51.529	49.096	40.482
Dominant stone diameter fraction	-18.378(-41.143-4.386)	0.115	-0.150	0.019	0.010	-0.018	51.531	49.094	40.287
Volume-weighted mean stone attenuation (HU)	-0.022(-0.051-0.006)	0.120	0.006	0.931	0.010	0.015	51.538	48.297	40.006
Aspect ratio of stone with largest estimated volume	9.214(-3.093-21.521)	0.144	0.090	0.157	0.009	0.003	51.568	48.572	39.531
Aspect ratio of stone with largest long diameter	8.478(-3.277-20.233)	0.159	0.108	0.091	0.008	-0.008	51.584	48.847	39.886
Lower calyx involvement	11.228(-4.618-27.074)	0.166			0.008	-0.017	51.591	49.065	40.028
Bilateral stones	14.215(-6.494-34.924)	0.180			0.007	-0.003	51.603	48.732	40.091
Stone volume distribution entropy	7.547(-3.692-18.786)	0.189	0.141	0.026	0.007	-0.019	51.611	49.123	40.556
Maximum stone attenuation (HU)	-0.015(-0.038-0.008)	0.193	-0.009	0.892	0.007	0.008	51.615	48.464	40.074
Aspect ratio of stone with largest estimated area	7.924(-4.169-20.018)	0.200	0.085	0.184	0.007	0.004	51.620	48.546	39.586
Maximum stone aspect ratio	6.353(-3.788-16.494)	0.221	0.125	0.050	0.006	-0.009	51.635	48.870	39.836
Area-weighted mean stone attenuation (HU)	-0.018(-0.047-0.011)	0.227	0.026	0.685	0.006	0.006	51.639	48.504	40.173
Stone number	1.294(-0.819-3.406)	0.231	0.171	0.007	0.006	-0.019	51.642	49.107	40.413
Multiple stones	8.756(-5.657-23.169)	0.235			0.006	-0.026	51.644	49.284	40.362
Volume-weighted mean aspect ratio	4.825(-9.236-18.887)	0.502	0.081	0.206	0.002	-0.005	51.746	48.778	40.023
Diameter-weighted mean stone attenuation (HU)	-0.009(-0.040-0.021)	0.542	0.062	0.328	0.002	-0.007	51.754	48.820	40.350
Normalized stone volume entropy	4.658(-14.091-23.407)	0.627	0.065	0.309	0.001	-0.019	51.769	49.112	40.511
Mean stone attenuation (HU)	0.000(-0.028-0.028)	0.985	0.061	0.343	0.000	-0.018	51.794	49.087	40.513

Table S11. Complete univariable linear regression analysis for operative time among stone-free episodes.

Variable	Beta (95% CI)	P	Spearman rho	Spearman P	Training R²	Validation R²	Training RMSE	Validation RMSE	Validation MAE
Volume-equivalent diameter (mm)	7.141(5.487-8.795)	<0.001	0.653	<0.001	0.329	0.447	37.111	30.845	22.530
Area-equivalent diameter (mm)	5.336(4.096-6.576)	<0.001	0.653	<0.001	0.328	0.481	37.155	29.882	21.546
Estimated total surface area (mm²)	0.061(0.047-0.075)	<0.001	0.655	<0.001	0.325	0.500	37.216	29.308	20.811
Total stone area (mm²)	0.216(0.164-0.267)	<0.001	0.653	<0.001	0.317	0.487	37.455	29.702	21.109
Estimated total stone volume (mm³)	0.026(0.020-0.033)	<0.001	0.653	<0.001	0.305	0.386	37.775	32.506	22.521
Length-only area-equivalent diameter (mm)	3.462(2.493-4.431)	<0.001	0.591	<0.001	0.251	0.388	39.203	32.438	23.340
Estimated surface area of stone with largest estimated area (mm²)	0.101(0.072-0.130)	<0.001	0.582	<0.001	0.243	0.279	39.435	35.216	23.629
Estimated surface area of stone with largest estimated volume (mm²)	0.101(0.072-0.130)	<0.001	0.581	<0.001	0.242	0.280	39.450	35.192	23.613
Estimated surface area of stone with largest long diameter (mm²)	0.101(0.072-0.130)	<0.001	0.576	<0.001	0.241	0.267	39.481	35.507	23.699
Estimated volume of stone with largest estimated area (mm³)	0.036(0.025-0.046)	<0.001	0.582	<0.001	0.236	0.175	39.609	37.661	24.464
Estimated volume of stone with largest estimated volume (mm³)	0.036(0.025-0.046)	<0.001	0.582	<0.001	0.235	0.175	39.619	37.674	24.467
Estimated volume of stone with largest long diameter (mm³)	0.036(0.025-0.046)	<0.001	0.573	<0.001	0.235	0.163	39.626	37.931	24.581
Estimated area of stone with largest estimated area (mm²)	0.345(0.241-0.448)	<0.001	0.577	<0.001	0.225	0.272	39.888	35.393	23.748
Estimated area of stone with largest estimated volume (mm²)	0.344(0.240-0.448)	<0.001	0.577	<0.001	0.224	0.274	39.901	35.331	23.717
Estimated area of stone with largest long diameter (mm²)	0.343(0.240-0.447)	<0.001	0.573	<0.001	0.224	0.266	39.903	35.529	23.755
Length-only volume-equivalent diameter (mm)	3.869(2.682-5.055)	<0.001	0.565	<0.001	0.219	0.330	40.052	33.944	23.813
Cumulative stone diameter (mm)	1.165(0.785-1.545)	<0.001	0.528	<0.001	0.198	0.351	40.571	33.412	24.277
Maximum stone diameter (mm)	3.503(2.149-4.856)	<0.001	0.495	<0.001	0.150	0.226	41.780	36.491	25.104
Long diameter of stone with largest estimated volume (mm)	3.446(2.099-4.792)	<0.001	0.490	<0.001	0.147	0.224	41.848	36.540	24.931
Long diameter of stone with largest estimated area (mm)	3.443(2.091-4.795)	<0.001	0.488	<0.001	0.146	0.218	41.877	36.669	24.982
Estimated surface-volume ratio	-53.014(-74.224--31.805)	<0.001	-0.513	<0.001	0.141	0.206	41.990	36.944	27.757
Staghorn stone	49.929(20.260-79.598)	0.001			0.069	0.078	43.711	39.819	30.012
Dominant stone volume fraction	-52.375(-86.264--18.485)	0.003	-0.250	0.002	0.059	0.084	43.950	39.684	29.974
Dominant stone area fraction	-45.516(-75.679--15.352)	0.004	-0.257	0.002	0.057	0.065	44.011	40.101	30.349
Dominant stone diameter fraction	-37.695(-63.963--11.426)	0.006	-0.289	<0.001	0.051	0.055	44.130	40.305	30.745
Stone volume distribution entropy	20.270(5.975-34.565)	0.006	0.254	0.002	0.050	0.080	44.157	39.780	30.170
Stone site count	12.855(3.779-21.931)	0.006	0.243	0.003	0.050	-0.058	44.159	42.661	32.312
Stone number	3.397(0.658-6.136)	0.016	0.271	<0.001	0.039	0.050	44.420	40.410	30.835

Variable	Beta (95% CI)	P	Spearman rho	Spearman P	Training R^2	Validation R^2	Training RMSE	Validation RMSE	Validation MAE
Bilateral stones	27.879(4.645-51.112)	0.020			0.037	0.001	44.475	41.453	30.854
Volume-weighted mean stone attenuation (HU)	-0.039(-0.072--0.006)	0.021	-0.041	0.623	0.036	0.027	44.487	40.909	31.132
Stone attenuation of stone with largest estimated volume (HU)	-0.031(-0.060--0.003)	0.032	-0.084	0.307	0.031	0.026	44.596	40.917	31.269
Area-weighted mean stone attenuation (HU)	-0.038(-0.072--0.003)	0.034	-0.034	0.683	0.030	0.018	44.615	41.094	31.303
Stone attenuation of stone with largest estimated area (HU)	-0.028(-0.056-0.000)	0.053	-0.063	0.445	0.025	0.025	44.732	40.939	31.271
Stone attenuation of stone with largest long diameter (HU)	-0.027(-0.055-0.001)	0.063	-0.041	0.623	0.024	0.010	44.774	41.263	31.682
Diameter-weighted mean stone attenuation (HU)	-0.032(-0.067-0.004)	0.081	0.001	0.987	0.021	0.000	44.838	41.477	31.699
Maximum stone attenuation (HU)	-0.023(-0.052-0.005)	0.108	-0.005	0.951	0.018	0.002	44.910	41.437	31.606
Normalized stone volume entropy	15.256(-5.477-35.990)	0.151	0.160	0.052	0.014	0.006	44.991	41.336	31.277
Mean stone attenuation (HU)	-0.022(-0.054-0.010)	0.183	0.013	0.879	0.012	-0.023	45.035	41.938	32.089
Multiple stones	9.744(-5.794-25.281)	0.221			0.010	-0.005	45.077	41.571	31.724
Volume-weighted mean aspect ratio	-9.387(-24.545-5.772)	0.227	-0.030	0.722	0.010	-0.041	45.083	42.317	33.197
Aspect ratio of stone with largest estimated area	-6.906(-20.596-6.785)	0.324	-0.039	0.634	0.007	-0.040	45.159	42.290	33.177
Aspect ratio of stone with largest long diameter	-6.405(-19.485-6.676)	0.339	-0.035	0.671	0.006	-0.032	45.168	42.135	32.614
Aspect ratio of stone with largest estimated volume	-6.465(-20.636-7.705)	0.373	-0.034	0.678	0.005	-0.039	45.186	42.262	33.102
Maximum stone aspect ratio	1.631(-10.320-13.583)	0.789	0.104	0.207	0.000	-0.025	45.299	41.978	32.158
Lower calyx involvement	0.921(-15.502-17.343)	0.913			0.000	-0.026	45.308	42.011	32.253

Table S12. Log-transformed operative-time sensitivity analysis.

Analysis	Variable	Beta (95% CI)	P	Spearman rho	Training R^2	Validation R^2	Validation RMSE	Validation MAE
Whole cohort	Volume-equivalent diameter (mm)	0.064 (0.054 to 0.073)	<0.001	0.678	0.413	0.445	0.442	0.348
Whole cohort	Area-equivalent diameter (mm)	0.048 (0.041 to 0.056)	<0.001	0.669	0.395	0.415	0.454	0.361
Whole cohort	Estimated total surface area (mm^2)	0.000 (0.000 to 0.000)	<0.001	0.669	0.289	0.280	0.503	0.406
Whole cohort	Estimated total stone volume (mm^3)	0.000 (0.000 to 0.000)	<0.001	0.678	0.222	0.210	0.527	0.427
Whole cohort	CSD (mm)	0.010 (0.008 to 0.013)	<0.001	0.530	0.196	0.180	0.537	0.433
Whole cohort	Maximum stone diameter (mm)	0.039 (0.032 to 0.046)	<0.001	0.591	0.332	0.367	0.472	0.367
Whole cohort	Staghorn stone	0.589 (0.414 to 0.764)	<0.001	0.372	0.150	0.124	0.555	0.439
Stone-free episodes	Volume-equivalent diameter (mm)	0.091 (0.073 to 0.109)	<0.001	0.653	0.405	0.448	0.414	0.318
Stone-free episodes	Area-equivalent diameter (mm)	0.068 (0.054 to 0.081)	<0.001	0.653	0.399	0.464	0.408	0.315
Stone-free episodes	Estimated total surface area (mm^2)	0.001 (0.001 to 0.001)	<0.001	0.655	0.357	0.429	0.421	0.326
Stone-free episodes	Estimated total stone volume (mm^3)	0.000 (0.000 to 0.000)	<0.001	0.653	0.319	0.343	0.452	0.351
Stone-free episodes	CSD (mm)	0.015 (0.010 to 0.019)	<0.001	0.528	0.235	0.297	0.467	0.365
Stone-free episodes	Maximum stone diameter (mm)	0.047 (0.032 to 0.062)	<0.001	0.495	0.206	0.267	0.477	0.369
Stone-free episodes	Staghorn stone	0.486 (0.142 to 0.830)	0.006	0.188	0.050	0.036	0.547	0.433

Table S13. Exploratory cutoff performance of representative stone burden metrics in the full cohort.

Variable	Cutoff rule	Cutoff	AUC	Sensitivity	Specificity	PPV	NPV	Accuracy	F1	Youden index
CSD (mm)	>=23.1	23.1 (optimal)	0.759	0.787	0.640	0.594	0.818	0.699	0.677	0.427
CSD (mm)	>=20	20 (manual)	0.759	0.844	0.512	0.536	0.831	0.645	0.656	0.356
CSD (mm)	>=25	25 (manual)	0.759	0.738	0.659	0.591	0.790	0.690	0.656	0.396
Area-equivalent diameter (mm)	>=13.213	13.213 (optimal)	0.767	0.787	0.640	0.594	0.818	0.699	0.677	0.427
Area-equivalent diameter (mm)	>=10	10 (manual)	0.767	0.894	0.351	0.479	0.831	0.568	0.624	0.244
Area-equivalent diameter (mm)	>=15	15 (manual)	0.767	0.660	0.735	0.624	0.764	0.705	0.641	0.394
Stone number	>=4.5	4.5 (optimal)	0.630	0.355	0.853	0.617	0.664	0.653	0.450	0.208
Stone number	>=4	4 (manual)	0.630	0.440	0.744	0.534	0.665	0.622	0.482	0.184
Stone number	>=5	5 (manual)	0.630	0.355	0.853	0.617	0.664	0.653	0.450	0.208
